# Intestinal CD169^+^ macrophages initiate mucosal inflammation by secreting CCL8 that recruits inflammatory monocytes

**DOI:** 10.1038/ncomms8802

**Published:** 2015-07-21

**Authors:** Kenichi Asano, Naomichi Takahashi, Mikiko Ushiki, Misa Monya, Fumiaki Aihara, Erika Kuboki, Shigetaka Moriyama, Mayumi Iida, Hiroshi Kitamura, Chun-Hong Qiu, Takashi Watanabe, Masato Tanaka

**Affiliations:** 1 Laboratory of Immune regulation, School of Life Science, Tokyo University of Pharmacy and Life Sciences, 1432-1 Horinouchi, Hachioji, Tokyo 192-0392, Japan; 2 Japan Science and Technology Agency, PRESTO, 4-1-8 Honcho, Kawaguchi, Saitama 332-0012, Japan; 3 Laboratory of Veterinary Physiology, School of Veterinary Medicine, Rakuno Gakuen University, 582 Bunkyodai-Midorimachi, Ebetsu, Hokkaido 069-8501, Japan; 4 Institute of Cell Biology, Shandong University School of Medicine, PO Box 73, No. 44 Wenhua Xi Road, Jinan, Shandong 250012, China; 5 Immunogenomics Laboratory, RIKEN Center for Integrated Medical Sciences, 1-7-22 Suehirocho, Tsurumi, Yokohama 227-0045, Japan

## Abstract

Lamina propria (LP) macrophages are constantly exposed to commensal bacteria, and are refractory to those antigens in an interleukin (IL)-10-dependent fashion. However, the mechanisms that discriminate hazardous invasion by bacteria from peaceful co-existence with them remain elusive. Here we show that CD169^+^ macrophages reside not at the villus tip, but at the bottom-end of the LP microenvironment. Following mucosal injury, the CD169^+^ macrophages recruit inflammatory monocytes by secreting CCL8. Selective depletion of CD169^+^ macrophages or administration of neutralizing anti-CCL8 antibody ameliorates the symptoms of experimentally induced colitis in mice. Collectively, we identify an LP-resident macrophage subset that links mucosal damage and inflammatory monocyte recruitment. Our results suggest that CD169^+^ macrophage-derived CCL8 serves as an emergency alert for the collapse of barrier defence, and is a promising target for the suppression of mucosal injury.

## Abstract (web summary)

Macrophages and dendritic cells residing in the lamina propria are involved in controlling mucosal immune balance. Here, the authors identify CD169^+^ macrophages as contributors to the inflammation of DSS colitis through their role in mediating the recruitment of monocytes by secreting the cytokine CCL8.

## Graphical abstract

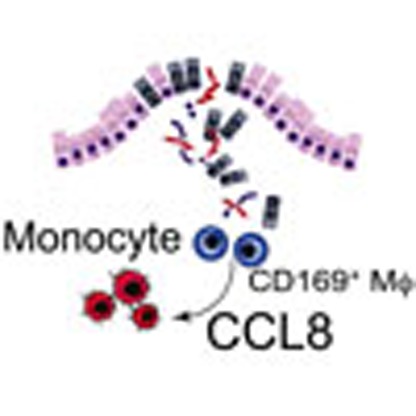

The intestine is the largest compartment of the immune system, and is lined by a single layer of epithelium that harbours trillions of commensal bacteria. Immune responses in the intestine are strictly tuned, where the ability to intercept invading pathogens must be balanced with the need to tolerate commensal bacteria. A yet unanswered question in mucosal immunology is how the immune system distinguishes pathogens from potentially beneficial commensals^1^,^2^.

Among the wide variety of immune cells, lamina propria (LP)-resident mononuclear phagocytes, mainly macrophages and dendritic cells (DCs), are the major contributors to the orchestration of mucosal immune balance^3^,^4^. They express an array of receptors that recognize both pathogen-associated molecular patterns and tissue damage to discriminate hazardous antigens from potentially beneficial ones. Macrophages and DCs in the intestine are heterogeneous in terms of origin, surface molecules and genetic markers^5^,^6^. For many years, there has been a lack of common criteria for reliably discriminating macrophages from other immune cells. The so-called ‘monocyte-waterfall' model was proposed recently and is emerging as the standard criterion for distinguishing resident macrophages from monocyte-derived ones according to the differential expression of CD64 and Ly6C^7^. CD64, mouse Fcγ receptor I, expression is restricted to resident macrophages, and is positively correlated with major histocompatibility complex class II and CX3CR1 expression and negatively correlated with Ly6C expression. It is also reported that LP macrophages can be subfractionated based on the expression of CX3CR1 (ref. 4). Classically, under the steady-state condition, LP macrophages and DCs can be divided into three subpopulations according to the expression patterns of CD11b and CD11c^4^. Although it is most likely that each subset plays a distinct role in the maintenance of gut homeostasis, the roles of different subsets in the regulation of mucosal immunity remain largely unknown.

Inflammatory bowel disease (IBD) is characterized by the chronic inflammation of the gastrointestinal tract^8^. The detailed aetiology of IBD in human and animal models remains to be elucidated. Nevertheless, it is widely accepted that the abnormal activation of immune cells towards microbiota or dietary antigen is critical to the exacerbation of inflammation. In human patients, genetic susceptibility as well as an imbalance in the composition of microbiota are associated with IBD^9^. In a mouse model of colitis, mucosal inflammation induces the robust accumulation of phagocytes that are derived from blood-borne monocytes. The high expression of Ly6C and the intermediate to low expression of CX3CR1 and CD64 are hallmarks of the infiltrating monocytes^7^,^10^,^11^,^12^. On recruitment to the inflammation site, Ly6C^hi^ macrophages give rise to pro-inflammatory phenotypes, producing cytokines, such as IL-6 and IL-23, to further activate Th17 cells and innate lymphoid cells. However, the cellular and molecular mechanisms that trigger the recruitment of those macrophages are largely unknown.

A subset of macrophages that express the CD169^+^ molecule on their surface and reside mainly in secondary lymphoid organs contribute to the regulation of immune response to cell-associated antigens^13^,^14^. In the marginal zone of the spleen, they capture apoptotic cells in the bloodstream and induce cell-associated antigen-specific tolerance^14^. A CD169^+^ counterpart in the lymph node sinus engulfs dead tumour cells that flow into the draining lymph node, and activates tumour antigen-specific CD8 T cells^13^. Those lines of evidence gave rise to the hypothesis that CD169^+^ macrophages serve as sentinels in immune organs that sense cell death, and either suppress or activate dead cell antigen-specific immune response.

Here we demonstrate that the selective depletion of CD169^+^ macrophages residing in LP ameliorates symptoms of dextran sodium sulfate (DSS)-induced colitis in mouse. Those macrophages show unique localization in a region distant from the epithelium–LP border. Microarray analysis revealed the upregulated expression of CCL8 exclusively by CD169^+^ macrophages under the inflammatory condition. Notably, the administration of neutralizing anti-CCL8 antibody improves the clinical symptoms of DSS-induced colitis in mouse. Our results highlight the pivotal role of LP-resident CD169^+^ macrophages in the progression of mucosal injury by producing CCL8.

## Results

### CD169-expressing macrophages in the intestine

**Figure 1:**
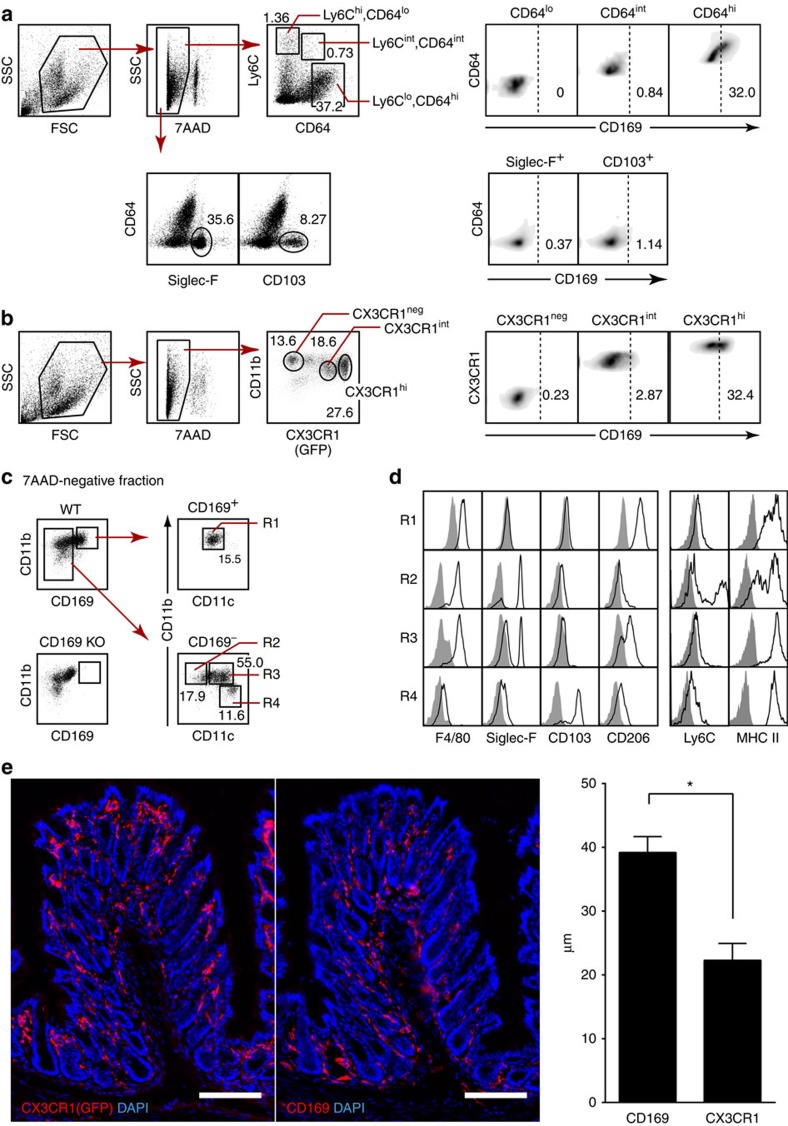
CD169^+^ macrophages in the lamina propria. (**a**) Flow cytometry of LP myeloid cells in the colon. CD11b^+^ and/or CD11c^+^ (‘myeloid') cells were enriched by magnetic sorting and stained for Ly6C, CD64, CD169 and Siglec-F or CD103. Dead cells were excluded by 7AAD staining. Numbers indicate frequencies of CD64^lo^, CD64^int^, CD64^hi^, Siglec-F^+^ and CD103^+^ fractions among 7AAD^−^ cells (left). Frequencies of CD169^+^ cells in each fraction are presented (right). Dashed lines represent isotype control. (**b**) Flow cytometry of LP myeloid cells in the colon of CX3CR1^gfp^ mice. Numbers indicate frequencies of CX3CR1^neg^, CX3CR1^int^ and CX3CR1^hi^ fractions among 7AAD^−^ myeloid cells (left). Frequencies of CD169^+^ cells in CX3CR1^neg^, CX3CR1^int^ and CX3CR1^hi^ fractions are presented (right). Dashed lines represent unstained control. (**a**,**b**) Representative of three independent experiments. (**c**) Subfractionation of LP myeloid cells in the colon according to the expression of CD11b and CD11c. Fifteen percent of the 7AAD^−^ myeloid cells expressed CD169 (top right). The specificity of anti-CD169 antibody was confirmed by staining LP myeloid cells of CD169 knockout (KO) mouse (bottom left). LP myeloid cells were subdivided into four subpopulations (R1–R4) based on the differential expression of CD169, CD11b and CD11c. Numbers indicate frequencies among 7AAD^−^ myeloid cells. Representative of five independent experiments. (**d**) Flow cytometry analysis of a panel of surface molecules on CD169^+^ and CD169^−^ cells in the colon. Shadow represents unstained control. Representative of two independent experiments. (**e**) CD169^+^ macrophages were localized distantly from the epithelial border. Consecutive colon sections from CX3CR1^gfp^ mice were stained for GFP (left) or CD169 (middle). Original magnification, × 20. Scale bars represent 100 μm. The distance of CX3CR1^+^ cells or CD169^+^ cells from the epithelial border was quantified (right). *P<0.05, Student's *t*-test. Representative of two independent experiments. DAPI, 4,6-diamidino-2-phenylindole.

Macrophages and DCs in the LP of the intestine are heterogeneous. Recently, CD64 was proposed as a reliable marker to discriminate resident macrophages in the gut from monocytes and DCs^7^,^15^. On the basis of the expression levels of CD64, macrophages and monocytes could be divided into CX3CR1^hi^, Ly6C^lo^, CD64^hi^-resident macrophages, CX3CR1^int^, Ly6C^hi^, CD64^lo^ monocytes and their intermediate. We examined CD169 expression on macrophages and DCs in the intestine using an anti-CD169 antibody having high specificity and sensitivity (clone M7) (Supplementary Fig. 1). As shown in Fig. 1a, CD169^+^ cells constituted 32% of Ly6C^lo^, CD64^hi^-resident macrophages in the colon. On the other hand, CD169^+^ cells were barely detected in the Ly6C^hi^, CD64^lo^ monocyte, Ly6^int^, CD64^int^ monocyte, Siglec-F^+^ eosinophil and CD103^+^ DC compartments. We also examined the presence of CD169^+^ macrophages in the small intestine. In agreement with the finding in the colon, CD169^+^ cells were detected only in the Ly6C^lo^, CD64^hi^ compartment, although their frequency (14.7%) was lower than that in the colon (Supplementary Fig. 2).

It is widely accepted that LP macrophages are also classified into subpopulations according to the expression level of CX3CR1, that is, CD11b^+^, CX3CR1^hi^ cells represent the resident macrophages, whereas CD11b^+^, CX3CR1^int^, Ly6C^hi^ cells accumulate in parallel with the development of inflammation^12^. Then, we examined the surface expression of CD169 on CX3CR1^hi^, CX3CR1^int^ and CX3CR1^neg^ cells in the colon using CX3CR1^gfp^ mice that express green fluorescent protein (GFP) under the control of the CX3CR1 promoter^16^. Approximately 30% of CX3CR1^hi^ cells expressed CD169, whereas <3% of CX3CR1^int^ and CX3CR1^neg^ cells were positive for CD169 (Fig. 1b). Therefore, the CD169^+^ macrophages can be categorized as a subpopulation of CX3CR1^hi^ macrophages in the colon.

We also examined a panel of surface molecules expressed on CD169^+^ cells and other cell types subfractionated by the expression of CD11b and CD11c^17^,^18^. We divided LP myeloid cells in the colon into four subpopulations: fraction R1: CD11b^+^, CD11c^dul^, CD169^+^ macrophages; fraction R2: CD169^−^, CD11b^+^, CD11c^−^ infiltrated cells; fraction R3: CD169^−^, CD11b^+^, CD11c^+^-resident cells; and fraction R4: CD169^−^, CD11b^−^, CD11c^hi^ DCs, and analysed the surface markers on these subpopulations. The analysis of CD169-deficient mice confirmed the specificity of the antibody (Fig. 1c, bottom left; Supplementary Fig. 1). Fifteen percent of 7AAD^−^, CD11b- and/or CD11c-positive cells, called ‘myeloid cells' hereafter, were CD169 positive. The CD169^+^ cells appeared to be homogeneous in terms of CD11b and CD11c expression levels (CD11b^+^ and CD11c^dul^) (Fig. 1c, top right). As shown in Fig. 1d and Supplementary Fig. 3, CD169^+^ cells in fraction R1 were distinct from those in the other three subpopulations. For instance, CD169^+^ cells showed high expression of CD206, whereas the other three subpopulations showed intermediate or low expression of this molecule. Collectively, these results further confirm that CD169^+^ macrophages are a subpopulation of LP-resident CX3CR1^hi^, CD64^hi^ macrophages.

We next examined the localization of CD169^+^ macrophages, and compared it with the localization of CX3CR1^+^ macrophages. Consistent with the report by Niess *et al.*^19^, CX3CR1^+^ (GFP^+^) cells resided evenly throughout the LP (Fig. 1e, left). In contrast, anti-CD169 antibody staining showed the preferential bias of CD169^+^ cell localization towards the area close to the muscularis mucosa (Fig. 1e, middle), in good agreement with the recent report demonstrating them surrounding the intestinal epithelial crypts^20^. The distance of CD169^+^ cells from the epithelial border was significantly larger than that of CX3CR1^+^ cells (Fig. 1e, right). Collectively, the unique localization and the surface phenotype of CD169^+^ macrophages suggest the specific role played by those cells in the regulation of mucosal immunity.

**Figure 2:**
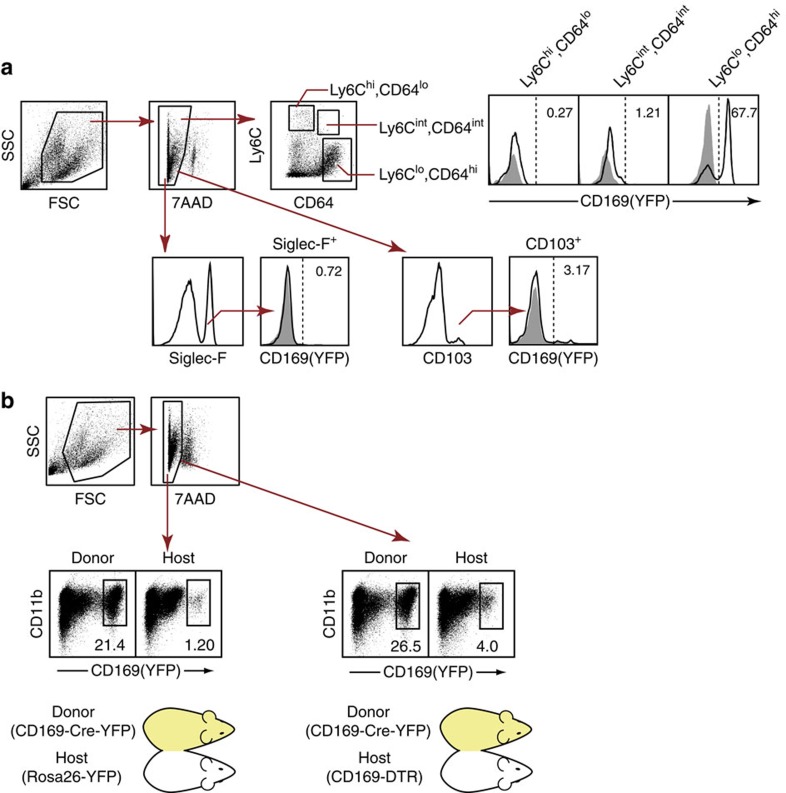
Monocyte-derived origin of gut CD169^+^ macrophages. (**a**) Expression of YFP in the LP myeloid cells from CD169-Cre-YFP reporter mice. LP myeloid cells in the colon were stained for Ly6C, CD64 and Siglec-F or CD103 and fractionated as in Fig. 1a. Numbers indicate frequencies of YFP^+^ cells among each fraction. Shadows indicate WT control. Representative of two independent experiments. (**b**) Rosa26-YFP mice (host, left) or CD169-DTR mice (host, right) were parabiotically joined with CD169-Cre-YFP mice (donor). DT was injected into CD169-Cre-YFP and CD169-DTR parabionts (right) 1 week before separation. LP myeloid cells were analysed by flow cytometry for the presence of YFP^+^ cells 5 weeks after initial cojoinment. Numbers indicate frequencies among 7AAD^−^ LP myeloid cells. Representatives of four pairs are shown.

It was reported that in the lymph node, not only subcapsular sinus macrophages but also innate-like lymphoid cells are stained for CD169 by flowcytometry^21^. Those lymphoid cells are in close contact with the subcapsular sinus macrophages and are speculated to acquire the CD169^+^ macrophage-derived membrane fragment. To distinguish CD169-expressing cells from those coated with the CD169^+^ cell-derived membrane fragment, we generated reporter mice that express yellow fluorescent protein (YFP) selectively in CD169^+^ cells and their descendants (CD169-Cre-YFP mice^22^). Gene recombination was detected in a wide variety of tissues including the LP of the intestine. In the colon and the small intestine, 67.7 and 57.1%, respectively, of Ly6C^lo^, CD64^hi^-resident macrophages expressed YFP. In contrast, YFP^+^ cells were not detected in the Ly6C^hi^, CD64^lo^ and Ly6C^int^, CD64^int^ monocyte fractions both in the colon and the small intestine (Fig. 2a; Supplementary Fig. 4a). The YFP-positive cells were CD11b^+^, CD11c^dul^, F4/80^+^ and CD206^+^ (Supplementary Fig. 4b), consistent with the result obtained by anti-CD169 antibody staining (Fig. 1d). Rosa 26-YFP mice not crossed with CD169-Cre mice do not express YFP (Supplementary Fig. 4b, bottom left). This result clearly demonstrates that the LP CD169^+^ cells indeed express CD169 molecules and are not other cell types that are simply coated with the CD169^+^ macrophage-derived membrane fragment. It was also revealed that the frequency of YFP^+^ cells among the Ly6C^lo^, CD64^hi^ compartment was higher than that of CD169^+^ cells (67 versus 32%, Figs 1a and 2a). This result suggests that part of YFP^+^ cells lost CD169 expression at some point during maturation, but those cells never differentiated into cell types other than resident macrophages.

**Figure 3:**
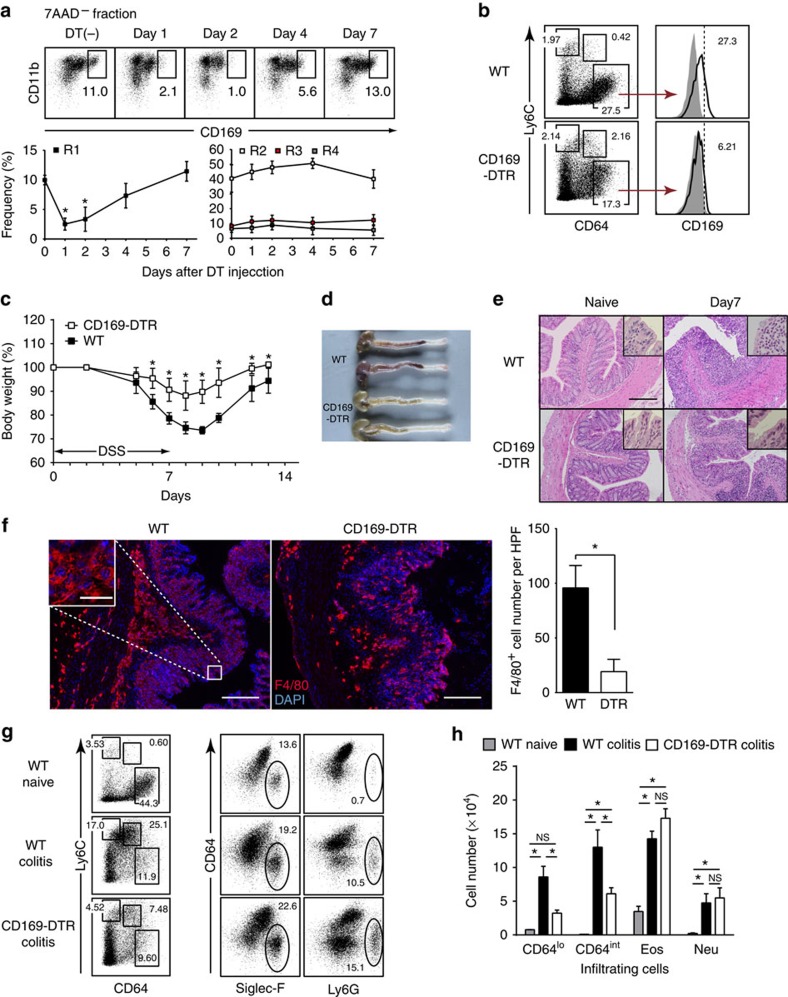
Amelioration of DSS-induced colitis in CD169-DTR mice. (**a**) Depletion and kinetics of recovery of CD169^+^ cells in the colon of DT-injected CD169-DTR mice. Top, numbers indicate frequencies of CD169^+^ cells among 7AAD^−^ myeloid cells enriched by magnetic sorting at indicated time points. Bottom, average frequencies of CD169^+^ (R1) and CD169^−^ cells (R2–R4, see Fig. 1c). Error bars mark s.d., *n*=3 mice, *P<0.05, one-way analysis of variance (ANOVA) with multiple comparison. (**b**) Injection of DT into CD169-DTR mice depletes CD64^hi^, CD169^+^ but not CD64^hi^, CD169^−^-resident macrophages. Left, numbers indicate frequencies of CD64^lo^ monocytes, CD64^int^ monocytes and CD64^hi^-resident macrophages among 7AAD^−^ myeloid cells. Right, frequencies of CD169^+^ cells among Ly6C^lo^, CD64^hi^-resident macrophages. Shadow, isotype control. (**c**) Depletion of CD169^+^ macrophages protects mice from DSS-induced colitis. DT was injected on days −1 and 3. The average body weight change and s.e.m. of 4–6 mice. *P<0.05, two-way ANOVA with multiple comparison. Representative data of five independent experiments. (**d**) Macroscopic observation of colons on day 7. Representative images of 12 mice. (**e**) Left, haematoxylin and eosin staining of paraffin sections from naive (left) or colitis (right) WT (top) or CD169-DTR mice (bottom). Loss of goblet cells in WT day 7, but not in CD169-DTR day 7 colon (insets). Scale bar, 100 μm. Original magnification, × 20. (**f**) F4/80 staining of the colon on DSS day 5. Original magnification, × 20. Scale bars, 200 μm. Inset, diffuse infiltration of F4/80^+^ cells in the villi of WT colon. Original magnification, × 40. Scale bar, 20 μm. Right, average numbers and s.d. of F4/80^+^cells per high-power field (HPF). *P<0.05, Student's *t*-test. Representative of two independent experiments. (**g**) Flow cytometry of colon myeloid cells from WT naive (top left), WT colitis (middle left) or CD169-DTR colitis (bottom left) mice. Numbers indicate frequencies among 7AAD^−^ myeloid cells. Representative data of four mice are shown. (**h**) Selective reduction of Ly6C^hi^, CD64^lo^ and Ly6C^int^, CD64^int^ monocytes in the colon LP of CD169-DTR colitis mice. Absolute numbers of infiltrating cells in **g**. Average numbers and s.d. of four mice. Eos: Siglec-F^+^ eosinophils, Neu: Ly6G^+^ neutrophils. *P<0.05, NS, not significant, one-way ANOVA. (**g**,**h**) Mice were injected with DT on days −1 and 3, and analysed on day 5.

Tissue macrophages are divided into two groups according to their origin^23^,^24^. The first population is derived from hematopoietic stem cells via peripheral blood monocytes. The second population of macrophages, such as brain microglia^25^,^26^ and skin Langerhans cells^27^,^28^, are distributed to the respective organs prior to definitive haematopoiesis. They replicate themselves locally throughout their lifespan^29^. To explore the origin of LP CD169^+^ macrophages, we investigated the presence of YFP^+^ cells in the colons of wild-type (WT) mice that are parabiotically joined with CD169-Cre-YFP mice. As shown in Fig. 2b, YFP^+^ cells were detected in the colons of WT mice demonstrating blood monocyte-derived origin of CD169^+^ macrophages, although their frequency (1.2%) was lower than that of CD169-Cre-YFP parabiont (21.4%) 5 weeks after the operation. The difference in the frequency of YFP^+^ cells between the two parabionts may have resulted from the slow turnover of those cells under the steady-state condition. To confirm this, we next joined CD169-diphtheria toxin receptor (DTR) mice, which express human DTR under the control of the CD169 promoter, with CD169-Cre-YFP mice. In the CD169-DTR mice, a single injection of diphtheria toxin (DT) induced the transient and selective depletion of CD169-expressing cells, which recovered 1 to 2 weeks after the DT injection^13^,^14^ (also see Fig. 3a,b). The depletion of CD169^+^ macrophages in CD169-DTR mice creates a cellular niche that may promote the rapid repopulation of those cells. Consistent with the hypothesis, the proportion of YFP^+^ cells was increased from 1.2 to 4% by DT injection (Fig. 2b). This finding indicates that at least part of CD169^+^ macrophages were derived from blood-borne monocytes, in agreement with the generally accepted concept that intestinal macrophages are constantly replenished by blood monocytes^10^,^30^. Collectively, we were able to comprehensively characterize CD169^+^ macrophages residing in the LP in terms of surface phenotype, localization and development. These findings prompted us to reveal their precise role in mucosal immunity.

### CD169^+^ macrophages promote DSS-induced colitis in mouse

The intestine is the largest barrier organ in the human body, and is important for the digestion and absorption of nutrients. Mucosal immunity is finely tuned to intercept pathogens as well as to maintain tolerance to dietary antigens and potentially beneficial commensal bacteria. We hypothesized that CD169^+^ macrophages localizing in the LP contribute to the regulation of mucosal immunity. To study the role of those macrophages, we utilized CD169-DTR mice in which CD169^+^ macrophages (R1) were transiently deleted by DT injection (Fig. 3a). The proportions of other myeloid cells (R2–R4) (Fig. 3a; Supplementary Fig. 5a) and lymphocytes (Supplementary Fig. 5b,c) were unaffected by the DT injection. We also confirmed that injection of DT depletes only CD64^hi^, CD169^+^ cells but not CD64^hi^, CD169^−^-resident macrophages in CD169-DTR mice (Fig. 3b).

The administration of DSS in drinking water induces epithelial injury that manifests as a clinical symptom of acute colitis. This model is considered innate immunity dependent, because colitis develops in lymphocyte-deficient mice. Then, we compared the severity of colitis between WT and CD169-DTR mice. Clinically, WT mice exhibited transient weight loss (Fig. 3c) and haemorrhagic diarrhoea. Grossly, the colons of WT mice were oedematous and filled with bloody stools (Fig. 3d, top). Remarkably, however, the symptoms of colitis were dramatically improved in CD169-DTR mice depleted of CD169^+^ cells by the DT injection on days −1 and 3 (Fig. 3c,d, bottom). We confirmed that a single DT injection on day −1 also significantly suppressed the DSS-induced colitis, although a slight decrease of body weight was seen late in the course of colitis (from day 9 to 14) compared with those injected with DT twice (Supplementary Fig. 6). This is most likely due to the recovery of CD169^+^ macrophages 7 days after the DT injection (Fig. 3a). Pathologically, the colons of WT mice were characterized by de-epithelialization of mucosa, loss of goblet cells and infiltration of a large number of immune cells in the LP (Fig. 3e, top right). Consistent with the clinical symptoms of DSS-induced colitis, the colons of DT-injected CD169-DTR mice were protected from those changes (Fig. 3e, bottom right). We examined the contribution of CD169^+^ macrophages in the development of another colitis model, T-cell-transfer colitis, which is induced by the adoptive transfer of WT naive CD4 T cells into lymphocyte-deficient mice. The injection of naive CD4 T cells into RAG1 knockout (KO) mice induced diarrhoea and weight loss in 4 weeks. The depletion of CD169^+^ macrophages in RAG1 KO mice that were crossed with CD169-DTR mice did not ameliorate the clinical symptoms of T-cell-transfer colitis (Supplementary Fig. 7). These results indicate that CD169^+^ macrophages play an important role in the pathology of innate immunity-dependent colitis, but not T-cell-transfer colitis. Thus, we analysed the function of CD169^+^ macrophages in the development of DSS-induced colitis in further experiments.

The administration of DSS to WT mice induces the accumulation of inflammatory cells, such as monocyte-derived macrophages, eosinophils and neutrophils, which further promotes mucosal inflammation. Thus, we compared the extent of infiltration between WT and CD169-DTR mice. Immunohistochemistry revealed diffuse infiltration of F4/80^+^ cells into the submucosa and the LP in WT mice (Fig. 3f, left). F4/80^+^cells were also detected in those regions in CD169-DTR mice, but to a lesser extent in the LP (Fig. 3f, middle and right). F4/80^+^ cells in the LP comprise monocytes, macrophages and eosinophils, and are undistinguishable by immunohistochemistry. To determine the composition of infiltrating cells, we enriched LP CD11b^+^ and/or CD11c^+^ cells of the colon by magnetic sorting and analysed them by flow cytometry. The frequencies of Ly6C^hi^, CD64^lo^ and Ly6C^int^, CD64^int^-infiltrating monocytes were lower, whereas the frequencies of CD64^−^, Siglec-F^+^ eosinophils and CD64^−^, Ly6G^+^ neutrophils were slightly higher in the colon of DSS-treated CD169-DTR mice than in that of WT mice (Fig. 3g). We next compared the absolute number of infiltrating cells, and found that the number of Ly6C^hi^, CD64^lo^ and Ly6C^int^, CD64^int^ monocytes was significantly reduced in CD169-DTR mice (Fig. 3h), whereas those of eosinophils (Eos) and neutrophils (Neu) was not statistically different between the two groups. These results strongly suggest that CD169^+^ macrophages promote the accumulation of monocyte-derived macrophages in the inflamed colon.

**Figure 4:**
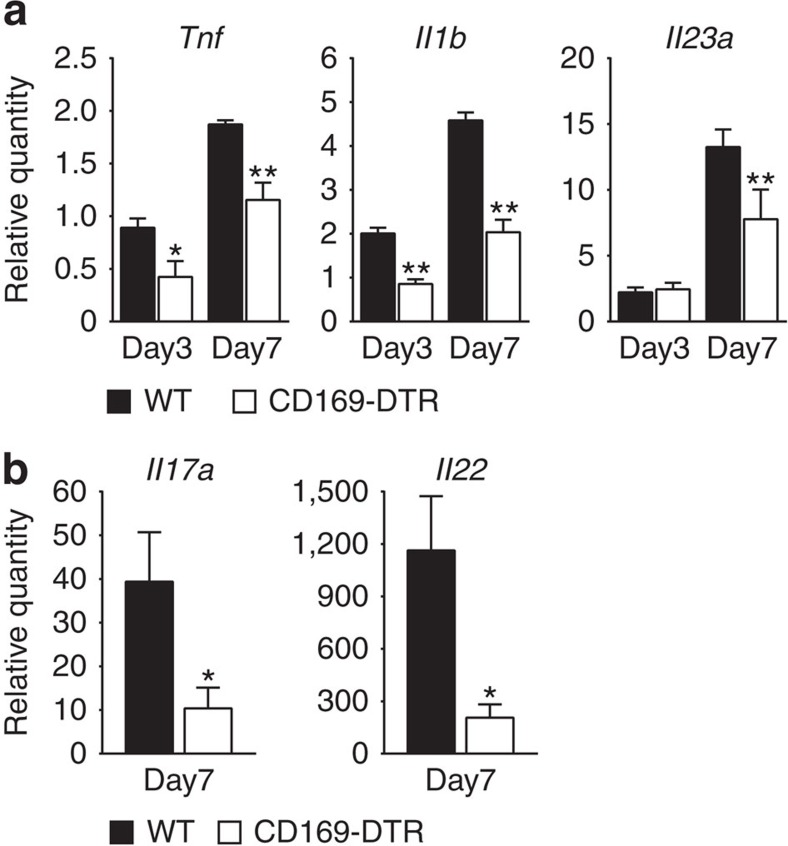
Immune cells are not fully activated in the absence of CD169^+^ macrophages. (**a**) Pro-inflammatory cytokine mRNA levels in LP myeloid cells from WT (black bar) or CD169-DTR (white bar) mice administered with 3.5% DSS for the indicated number of days were determined by qRT–PCR. (**b**) IL-17 or IL-22 mRNA levels in colon tissues from WT (black bar) or CD169-DTR (white bar) mice that received DSS for 7 days were determined by qRT–PCR. Expression levels were calculated as relative amounts normalized to beta actin and shown as fold induction compared with the expression in WT naive myeloid cells (**a**) or colon (**b**). *n*=4 (**a**) or 10 (**b**). Average values are shown with s.d.*P<0.05, **P<0.01, Student's *t*-test.

We next hypothesized that the infiltrated immune cells were not fully activated in the absence of CD169^+^ macrophages. It is considered that pro-inflammatory cytokines, such as TNFα, IL-6 and IL-23, produced by innate immune cells promote and sustain chronic mucosal inflammation in co-ordination with effector cytokines, such as IL-17 and IL-22, produced by lymphocytes in both mouse and human IBD^31^. To assess the activation state of inflammatory cells in the LP, we quantified TNFα, IL-1β and IL-23 messenger RNA (mRNA) expression in CD11b^+^ and/or CD11c^+^ cells. Quantitative reverse transcription–PCR (qRT–PCR) analysis revealed reduced cytokine production by those cell types in CD169^+^ macrophage-depleted mice (Fig. 4a). IL-17 and IL-22 mRNA expression in colon tissue was also significantly decreased in CD169^+^ macrophage-depleted mice (Fig. 4b). The precise roles of IL-17-producing T helper (Th17) family cytokines in colitis remain controversial^32^; nevertheless, those cytokines are thought to be correlated with the overall activity of mucosal inflammation. It was reported that those cytokines are produced not only by CD4^+^, Th17 cells, but also by group-3 innate lymphoid (ILC3) cells^33^,^34^,^35^,^36^ and γδT cells^37^. To examine whether the frequencies of those cells are decreased in the absence of CD169^+^ macrophages, we crossed our CD169-DTR mice with RORγt^gfp^ mice in which the gene encoding GFP was targeted to the RORγt gene locus^38^. Flow cytometry showed that the frequencies of Th17 cells (CD3^+^, CD4^+^, GFP^+^), ILC3 cells (CD3^−^, B220^−^, NKp46^+^, GFP^+^), LTi cells (CD3^−^, B220^−^, NKp46^−^, GFP^+^)^36^ and γδT cells (CD3^+^, B220^−^, TCRγδ^+^, GFP^+^) did not change with the depletion of CD169^+^ macrophages (Supplementary Fig. 8). This result indicates that the decreased IL-17 and IL-22 mRNA expression in CD169-DTR mice was not due to the disappearance of effector cytokine producers, but might have resulted from the suppressed inflammation of the colon. Collectively, our data highlight the role of CD169^+^ macrophages in the promotion of DSS-induced colitis.

### Selective expression of CCL8 by CD169^+^ macrophages in colitis

**Figure 5:**
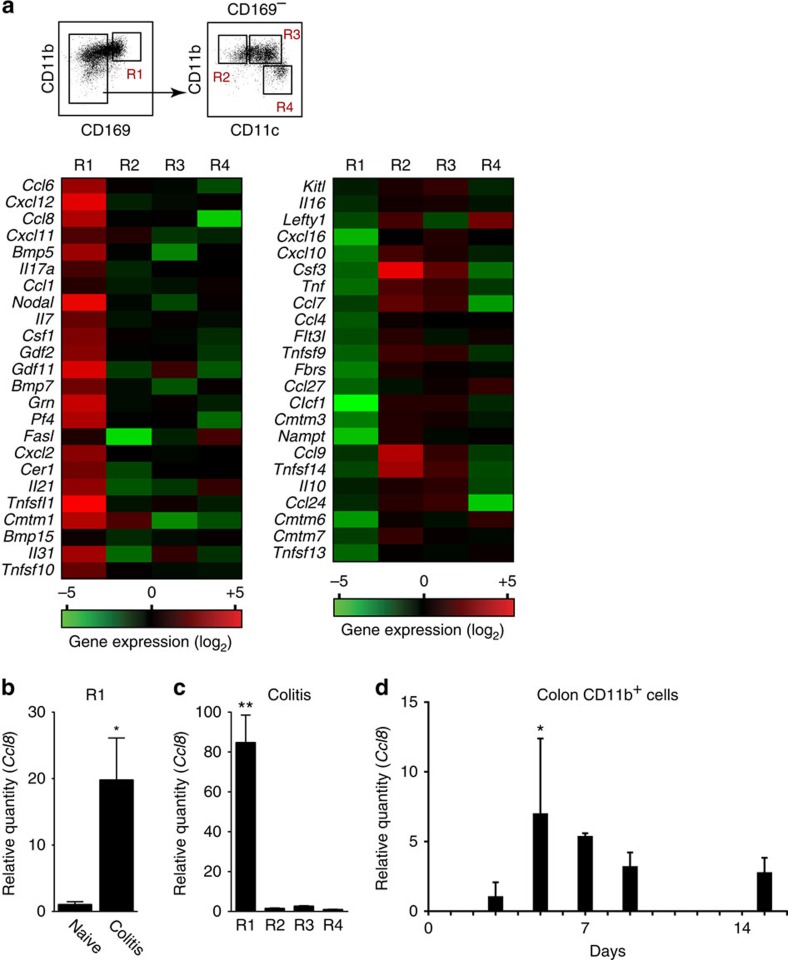
Expression of CCL8 mRNA by CD169^+^ macrophages under inflammatory condition. (**a**) CD169^+^ and CD169^−^ myeloid cells were purified from the LP of WT naive and DSS-induced colitis mice (top) by a cell sorter. Total RNA extracted from fractionated myeloid cells was hybridized to the Affimetrix Mouse Genome 430 2.0 Array chip. Heat map of mRNA upregulated (bottom left) or downregulated (bottom right) in CD169^+^ macrophages (R1) of colitis mice relative to their expression in CD169^+^ macrophages of naive mice. Expression levels are displayed as fold induction over naive controls (log_2_). (**b**) Upregulated expression of CCL8 mRNA in CD169^+^ macrophages (R1) 5 days after DSS administration was validated by qRT–PCR. (**c**) CCL8 mRNA expression 5 days after DSS administration was selectively upregulated in CD169^+^ cells. Values are presented as fold induction compared with the expression levels in naive CD169^+^ macrophages (**b**) or cells in R4 (**c**). Average values and s.d. of triplicate experiment are shown. *P<0.05, Student's *t*-test, **P<0.01, one-way analysis of variance (ANOVA). (**d**) CCL8 mRNA expression is upregulated in response to DSS administration in LP CD11b^+^ cells. LP CD11b^+^ cells in the colon were enriched by magnetic sorting from mice administered with DSS. CCL8 mRNA expression at the indicated time point was quantified by qRT–PCR and expressed as fold induction compared with naive CD11b^+^ cells. Data are representative of two independent experiments. *P<0.05, one-way ANOVA.

The results derived from the DSS-induced colitis mouse model suggest that CD169^+^ macrophages produce signalling molecules that promote CD169^−^, Ly6C^hi^ macrophage accumulation. To identify such molecules, we globally compared the mRNA expression profiles of fractionated CD169^+^ macrophages (R1) and CD169^−^ cells (R2–R4) (Fig. 5a, top) from naive and colitis mice. Among the mRNAs upregulated or downregulated in CD169^+^ macrophages (Fig. 5a, bottom), we focused on cytokine genes whose mRNA expression was increased by more than 5-fold in CD169^+^ macrophages of colitis mice compared with naive mice. qRT–PCR analysis confirmed that among those cytokines, CCL8 mRNA was upregulated by 20-fold in CD169^+^ macrophages under the inflammatory condition (Fig. 5b), but not in the other cell types (Fig. 5c). Its expression was upregulated early in the course of colitis, and was gradually decreased after the removal of DSS (Fig. 5d). Although microarray analysis revealed the upregulated expression of other chemokine genes, such as *CCL6*, *CXCL2* and *PF4*, we could not validate their expression 5 days after the DSS administration and therefore, did not study them further in the current project.

### CCL8 production in response to sterile and non-sterile stimuli

**Figure 6:**
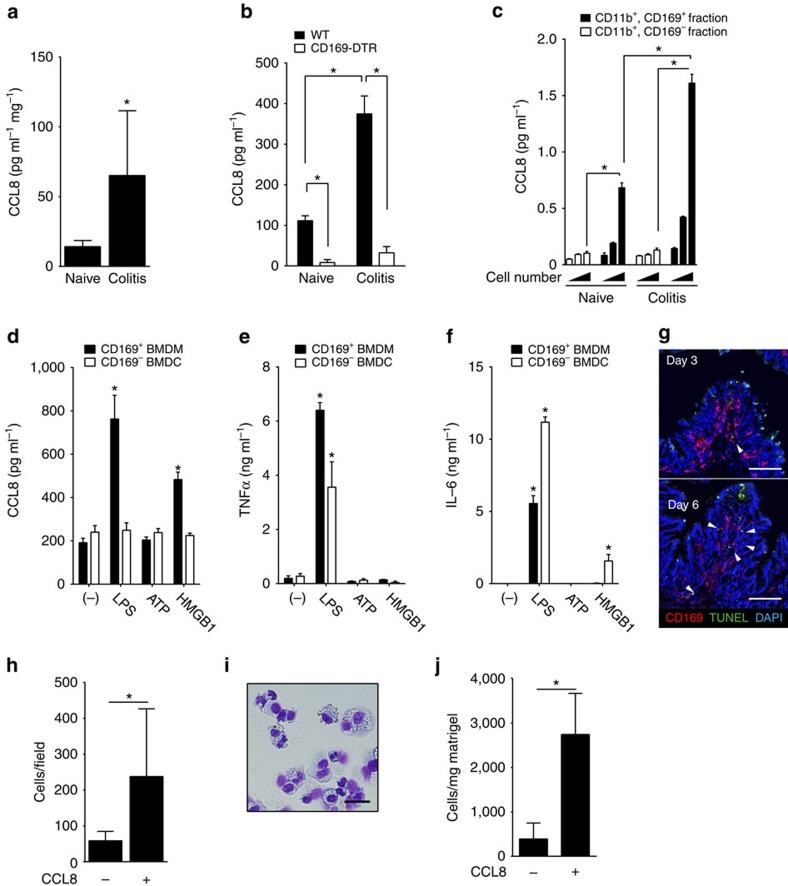
CCL8 is exclusively produced by CD169^+^ macrophages in response to sterile and non-sterile inflammatory stimuli. (**a**) CCL8 concentration in the culture medium of colon explant was quantified by ELISA and divided by the dry weight of the explant. Average and s.d. of three mice. Representative data from two independent experiments. *P<0.05, Student's *t*-test. (**b**) LP myeloid cells from the colon of indicated mice were cultured overnight *in vitro*. CCL8 concentrations in the culture medium were quantified. Average and s.d. of three mice. *P<0.05, two-way analysis of variance (ANOVA). (**c**) CD11b^+^, CD169^+^ and CD11b^+^, CD169^−^ cells from the colon of WT naive or colitis mice were fractionated by a cell sorter. Different numbers of fractionated macrophages were cultured *in vitro.* CCL8 concentrations in the culture supernatant were quantified. Representative data from two independent experiments. Error bar marks s.d. *P<0.05, two-way ANOVA. (**d**–**f**) Bone marrow (BM) cells were cultured for 5 days in the presence of M-CSF (black bar) or GM-CSF (white bar) to induce or not to induce CD169 expression. Those cells were treated *in vitro* with various stimulants for 24 h. CCL8 (**d**), TNFα (**e**) and IL-6 (**f**) concentrations in the culture medium. Average values and s.d. of a triplicate experiment. Statistical differences relative to unstimulated BM cells (−) were determined. *P<0.05, Student's *t*-test. Representative data of two independent experiments. (**g**) Immunohistochemistry of colon sections from DSS-fed WT mice. Arrowheads indicate contact of CD169^+^ cells with TUNEL^+^ cells. Original magnification, × 20. Scale bars, 100 μm. (**h**) CCL8 attracts monocytic WEHI-3 cells *in vitro*. WEHI-3 cells were seeded in the upper chamber, whereas the lower chamber was supplied with serum-free medium with or without 100 nM CCL8. The number of cells in the lower chamber at 4 h was enumerated. Average values and s.d. of triplicate experiment. *P<0.05, Student's *t*-test. Data are representative of three independent experiments. (**i**) Cells that migrated towards CCL8 *in vivo* showed mononuclear morphology. Cells retrieved from the Matrigel plugs supplemented with CCL8 were cytospun onto a glass slide, and stained with modified Giemsa. Scale bar, 10 μm. (**j**) The number of cells per milligram Matrigel plug was quantified by flow cytometry. Average values and s.d. of three mice. *P<0.05, Student's *t*-test. Data are representative of three independent experiments. DAPI, 4,6-diamidino-2-phenylindole.

We further tried to examine CCL8 protein levels in the DSS-induced colitis mouse. For this purpose, we constructed an enzyme-linked immunosorbent assay (ELISA) that specifically detects mouse CCL8. The standard dose–response curve of our ELISA exhibited a linear shape in the concentration range of 50 pg ml^−1^ to 2 ng ml^−1^ (Supplementary Fig. 9). Using this ELISA, we found that the amount of CCL8 in the culture medium of colon explant resected from the colitis mouse was increased by more than 3-fold compared with that of naive colon (Fig. 6a). We next quantified the amount of CCL8 in the culture medium of LP myeloid cells purified from either naive or inflamed colon. As shown in Fig. 6b, we found relatively large amounts of CCL8 produced by LP myeloid cells of WT naive mice. The mucosal injury induced by DSS enhanced CCL8 production by WT myeloid cells (Fig. 6b). Strikingly, however, CCL8 was almost undetectable in the culture medium of CD169^+^ cell-depleted myeloid cells under both naive and inflammatory conditions (Fig. 6b). This result strongly indicates that CCL8 was produced by CD169^+^ macrophages in the colon LP. To confirm this, we quantified CCL8 concentrations in the culture medium of CD11b^+^, CD169^+^ and CD11b^+^, CD169^−^ cells fractionated by a cell sorter. Consistent with the result obtained by qRT–PCR (Fig. 5c), ELISA revealed exclusive CCL8 production by CD169^+^ macrophages and not by other myeloid cells, which was further enhanced by DSS administration (Fig. 6c). We also compared CCL8 production by fractionated Ly6C^lo^, CD64^hi^, CD169^+^ and Ly6C^lo^, CD64^hi^, CD169^−^-resident macrophages (Supplementary Fig. 10a,b). CCL8 was produced only by CD64^hi^, CD169^+^ fraction both under naive and the inflammatory condition. We further quantified other pro- and anti-inflammatory cytokine mRNA expression levels in the fractionated Ly6C^lo^, CD64^hi^, CD169^+^ and Ly6C^lo^, CD64^hi^, CD169^−^ macrophages by qRT–PCR. The expression levels of IL-1β, IL-6 and IL-23 were higher in the CD169^−^ fraction than the CD169^+^ fraction, whereas IL-10 expression was similar in both fractions (Supplementary Fig. 10c). This result suggests that unlike previously known cytokines that modulate mucosal inflammation, CCL8 is produced mainly by CD169^+^ macrophages in the colon.

Next, we explored the stimuli for CCL8 production by CD169^+^ macrophages. DSS-induced colitis is thought to be initiated by the destruction of the epithelial barrier and the subsequent influx of commensals into the LP, but the precise mechanism that triggers mucosal inflammation remains poorly defined. We first stimulated magnetically sorted LP myeloid cells *in vitro* and quantified the amount of CCL8 in the culture media. However, the cells were refractory to any stimuli, including Toll-like receptor (TLR) ligands and HMGB1. The unresponsiveness of the LP myeloid cells may have resulted from the loss of viability after intensive enzymatic treatments, and/or from IL-10-dependent tolerance to commensal bacteria^18^. To overcome this problem, we used CD169^+^ macrophages expanded *in vitro* from bone marrow (BM) precursor cells. We found that BM cells homogeneously express CD169 when cultured in the presence of the macrophage-colony stimulating factor (M-CSF), whereas culture in the presence of granulocyte macrophage (GM)-CSF induces CD169^−^ BM-derived dendritic cells (BMDCs)^22^. When these cells were stimulated with a TLR-agonist, lipopolysaccharide (LPS) or a damage-associated molecule, HMGB1, CD169^+^ BM-derived macrophages (BMDMs), and not CD169^−^ BMDC, produced CCL8 (Fig. 6d). Notably, IL-6 and TNFα production by CD169^−^ BMDC in response to the same stimulants was intact (Fig. 6e,f), which excluded the possibility that CD169^−^ BMDCs used in this assay were less viable than CD169^+^ BMDM. These results suggest that CCL8 production could be triggered by both sterile and non-sterile inflammatory stimuli. DSS-induced epithelial cell injury might be followed by the phagocytosis of dead cell corpses by the surrounding phagocytes. Thus, we wondered whether CD169^+^ macrophages directly engage with dead epithelial cells under the inflammatory condition. Immunohistochemistry of the colon sections of mice that were administered with DSS revealed only a small number of TUNEL^+^ cells on day 3, most of which were detected at the tips of villi (Fig. 6g, top). This result is compatible with a report that dead epithelial cells are disposed into the gut lumen in rat and mouse under physiological conditions^39^. However, DSS administration for 6 days induced further destruction of the epithelium and cell death, which allowed CD169^+^ macrophages to come into direct contact with dead cells (Fig. 6g, bottom). This observation raised the possibility that intestinal CD169^+^ macrophages respond to dead cell-derived molecules subsequent to progressive mucosal injury *in vivo*.

### CCL8 recruits inflammatory monocyte-derived macrophages

The results derived from the DSS-induced colitis model mouse suggested the chemoattractive effect of CCL8 on monocytes and macrophages (Fig. 3g,h). Therefore, the migration of the monocytic cell line, WEHI-3 cells, towards CCL8 was examined first *in vitro*. Using a transwell chamber, we found that WEHI-3 cells migrated towards CCL8 (Fig. 6h). To assess the chemoattractive effect of CCL8 *in vivo*, we subcutaneously injected WT mice with Matrigel alone or Matrigel supplemented with CCL8. At 24 h after injection, the infiltrated cells were retrieved from the Matrigel plugs, cytospun on a glass slide and stained with modified Giemsa. Grossly, the Matrigel plugs without CCL8 were transparent, whereas those containing CCL8 were cloudy. A majority of the infiltrated cells were mononuclear (Fig. 6i), indicating the preferential recruitment of monocytes and/or macrophages by CCL8. The number of cells retrieved from the Matrigel plugs was significantly larger in the plugs supplemented with CCL8 (Fig. 6j). Collectively, the results demonstrate that CCL8 attracts monocyte-derived macrophages.

### Amelioration of DSS-induced colitis by blockade of CCL8

**Figure 7:**
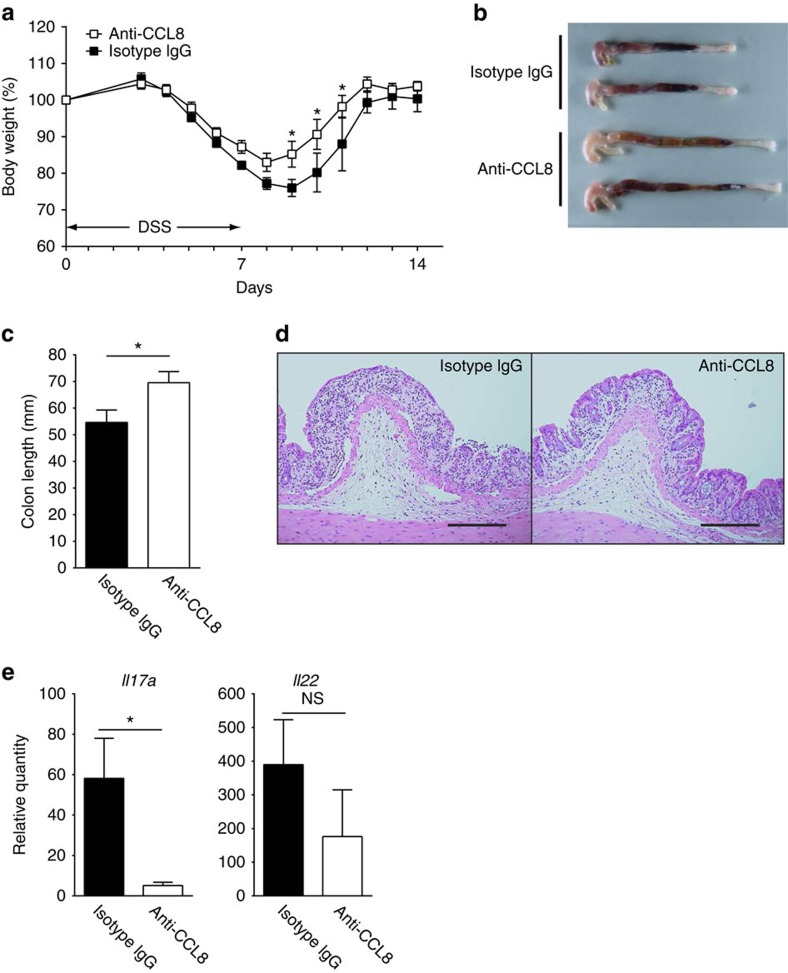
Amelioration of DSS-induced colitis by anti-CCL8-neutralizing antibody. (**a**) WT mice were administered with 3% DSS in drinking water for 7 days. One hundred micrograms of anti-CCL8 antibody (clone 17D6, white squares) or isotype IgG (black squares) was injected intravenously into the mice on days 3 and 4. Body weight change relative to the initial value was plotted up to 14 days after the DSS administration. Values are averages and s.e.m. of four to six mice per group. Representative data of four independent experiments are shown. *P<0.05, two-way analysis of variance with multiple comparison. (**b**) Macroscopic observation of the colon 7 days after the administration of DSS from WT mice injected with isotype IgG (top two colons) or anti-CCL8 antibody (bottom two colons). (**c**) Average lengths and s.d. of four colons are shown. *P<0.05, Student's *t*-test. (**d**) Haematoxylin and eosin staining of colon section from WT mice that received DSS for 5 days. The mice were treated with isotype IgG (left) or anti-CCL8 antibody (right). Representative images of four mice per group are shown. Scale bar, 200 μm. Original magnification, × 20. (**e**) IL-17 and IL-22 mRNA expression levels in colon tissue of WT mice on day 7, which were treated with isotype IgG (black bar) or anti-CCL8 antibody (white bar) were determined by qRT–PCR. Expression levels are shown as fold induction relative to the expression level in WT naive colon. Average values and s.e.m. of four colons are shown. *P*<0.05. NS, not significant, Student's *t*-test. Representative data of three independent experiments are shown.

The identification of CCL8 as a cytokine produced exclusively by CD169^+^ macrophages under the inflammatory condition prompted us to neutralize this molecule *in vivo* in colitis mice. To this end, we generated anti-CCL8 antibody by immunizing Wistar rats with CCL8 peptide. To suppress the activity of CCL8, we intravenously injected one of these antibodies (clone 17D6) into WT mice on days 3 and 4 after the administration of DSS. WT mice injected with isotype immunoglobulin (Ig)G exhibited weight loss that bottomed 8 to 9 days after the administration of DSS (Fig. 7a). In contrast, anti-CCL8 antibody ameliorated the clinical symptoms of DSS-induced colitis (Fig. 7a). Macroscopically, anti-CCL8 antibody suppressed colorectal bleeding and shortening of the colon (Fig. 7b,c). Pathological examination revealed de-epithelialized mucosa with distorted crypts in the isotype IgG-injected mice, in contrast to the significantly reduced tissue damage in the anti-CCL8 antibody-injected mice (Fig. 7d). qRT–PCR revealed decreased IL-17 mRNA levels in mice treated with anti-CCL8 antibody (Fig. 7e). These results highlight the clinical importance of CD169^+^ macrophage-derived CCL8 in the progression of mucosal inflammation subsequent to epithelial injury.

## Discussion

Previous studies have described the roles played by intestinal macrophages and DCs in the induction of mucosal immunity and tolerance^4^,^40^. However, the phenotype, and more importantly, the function in the regulation of mucosal immunity of different macrophages are poorly understood, even though macrophages constitute a major subset of intestinal myeloid cells^41^. This study yielded several surprising findings. First, we comprehensively characterized LP CD169^+^ macrophages in terms of both surface phenotype and localization. The parabiosis experiment demonstrated the BM origin, at least in part, of this subset. Second, the selective depletion of CD169^+^ macrophages ameliorated symptoms of DSS-induced colitis in mouse. This phenotype was attributable to the reduced accumulation of colitis-associated CD169^−^, Ly6C^hi^, CD64^lo^ monocytes in the LP. Finally, we found that CD169^+^ macrophages produced CCL8 in response to epithelial injury. Treatment with anti-CCL8-neutralizing antibody suppressed clinical symptoms of DSS-induced colitis. Together these data identify CD169^+^ macrophages as the sentinel located distantly from the epithelial border, and CCL8 as a critical regulator of mucosal inflammation.

DSS-fed mice display severe inflammation in the absence of lymphocytes^42^,^43^. This demonstrates that innate immune cells are sufficient for disease onset and development. Thus, DSS-induced colitis is a useful model to explore therapies that regulate innate inflammatory responses^44^. However, DSS-induced colitis does not manifest the clinical course of human ulcerative colitis that is exacerbated followed by periods of remission. Pathologically, de-epithelialization precedes mucosal inflammation in DSS-induced colitis, which is not the case with human IBD. It should be noted that findings from this model cannot directly be extrapolated to human disease of IBD.

CD169^+^ macrophages are strategically located at the interface of lymphoid organs and bloodstream or lymphatic flow. The unique localization enables them to survey and engulf blood or lymph-borne particulate antigens, and induce immune tolerance to or activation towards those antigens in the spleen and lymph nodes^13^,^14^. We found that CD169^+^ macrophages show unique localization as well in the LP microenvironment; that is, they are located relatively distant from the boundary of the lumen and mucosa. To maintain homeostasis, the gut immune system adopts several active tolerogenic strategies for commensal microbes. One of those strategies is the hyporesponsiveness of macrophages and DCs to pathogen-associated molecular patterns expressed by commensal bacteria^1^,^18^. In fact, in contrast to phagocytes in the other organs, LP-resident macrophages are refractory to gut microbiota-derived TLR agonists and constitutively express anti-inflammatory cytokine IL-10 (refs 3, 18, 45, 46). In addition to commensal bacteria, phagocytes are constantly exposed to dead epithelial cells at the tips of villi due to the rapid turnover of those cells^39^,^47^. In this context, CD169^−^, CX3CR1^+^ phagocytes localizing at the boundary of the mucosa and lumen tend to be refractory to microbe products and endogenous adjuvants released by dead cells. In stark contrast, CD169^+^ macrophages are strategically located away from the perimeter, where they would not be constantly exposed to commensal bacteria and dead epithelial cells under physiological conditions. Instead, their adjacency to vascular-rich submucosa may allow them to directly release CCL8 into the systemic circulation. This finding prompted us to hypothesize that CD169^+^ macrophages respond to the collapse of frontline defence; once epithelial cells are injured pathologically, CD169^+^ macrophages come into contact with bacterium- and dead cell-derived components, and dictate the responses of other immune cells including inflammatory Ly6C^hi^, CD64^lo^ monocytes by producing CCL8. From this point of view, CD169^+^ macrophages and their CCL8 may offer an emergency alert system when gut frontline defence is under profound threat.

The present study showed the exclusive production of CCL8 by CD169^+^ macrophages in both LP and BM. *In vitro*, CCL8 was produced not by CD169^−^ BMDC but by CD169^+^ BMDM in response to stimulation with LPS and HMGB1. Although CD169^+^ macrophages in the LP may differ markedly from their CD169^+^ counterpart in the BM, these data suggest that CCL8 production is regulated by a CD169^+^ macrophage-specific pathway. Some inflammatory cytokines, such as TNFα and IL-6, were produced in comparable amounts by both phagocytes in response to the same stimulants. These results suggest that CCL8 does not belong to the ‘generic' pro-inflammatory cytokines, but is a chemokine armed with a specific aim: alert for the collapse of the mucosal barrier. CCL8 is a member of the CC-chemokine family that attracts monocytes and lymphocytes in human. However, the biological role, expression mechanism and cellular source of CCL8 under physiological or pathological conditions are poorly understood. It was reported that CCL8 is highly expressed in the skin, and recruits IL-5-producing CD4T helper cells to promote atopic dermatitis^48^, although the source of CCL8 was not identified in that work. Recently, it was suggested that keratinocytes in hair follicles produce CCL8 in response to mechanical stress to negatively regulate the influx of Langerhans cells, which may be important for the protection of the basal area of hair follicles^49^. Plasma CCL8 concentration is positively correlated with the severity of graft-versus-host disease in both mouse^50^ and human^51^. Those studies suggest that CCL8 regulates the trafficking of immune cells in barrier organs, and that CCL8 serves as a biomarker for inflammatory diseases. The roles of CCL8 in the regulation of mucosal inflammation are poorly defined. Immunohistochemical study of biopsy specimens revealed the upregulated expression of CCL8 in patients in the active phase of IBD^52^. This study supports the hypothesis that the degree of mucosal inflammation is dependent on the local expression of CCL8.

In the present study, the administration of anti-CCL8 antibody did not completely suppress the symptoms of DSS-induced colitis as compared with the depletion of CD169^+^ macrophages. CD169^+^ macrophages may express other molecules implicated in the progression of DSS-induced colitis. Alternatively, neutralization of CCL8 by anti-CCL8 antibody might be insufficient *in vivo*. At any rate, the roles and functions of CD169^+^ macrophages and CCL8 should be exploited further to develop a reliable strategy for the suppression of mucosal injury. In conclusion, we found that the CD169^+^ macrophage–CCL8 axis is a critical regulator of epithelial injury-induced mucosal inflammation.

## Methods

### Animals

C57BL/6J mice were purchased from CLEA Japan (Tokyo, Japan). CD169-DTR mice were generated in our laboratory. RORγt^gfp^ mice^38^ were kindly provided by D. Littman (New York University School of Medicine, USA). CX3CR1^gfp^ mice^16^ and ROSA26-YFP mice^53^ were kindly provided by D. Littman and S. Jung (Weizmann Institute of Science, Israel), and F. Costantini (Columbia University, USA), respectively, and maintained under specific pathogen-free conditions. CD169-Cre mice that harbour the improved *Cre* gene^54^ in the *Siglec1* gene (encoding CD169) locus were generated in our laboratory. CD169-Cre mice were crossed with ROSA26-YFP reporter mice to visualize CD169^+^ cells (CD169-Cre-YFP mice^22^). RORγt^gfp^ mice, CX3CR1^gfp^ mice or RAG1-deficient (KO) mice were crossed with CD169-DTR mice. All experiments using mice were approved by the Tokyo University of Pharmacy and Life Sciences Animal Care Committee or RIKEN, RCAI Animal Use Committee, and performed in accordance with applicable guidelines and regulations.

### Generation of monoclonal antibodies

To generate anti-CD169 antibody, HEK293T cells that express CD169 molecules were injected intraperitoneally three times into Wistar rats. TiterMax Gold (TiterMax, GA) was also injected at the initial immunization. Splenocytes were fused with NSO^bcl2^ myeloma cells^55^ by PEG1500 (Roche, Germany). Hybridoma cells were selected in DMEM/10% FCS containing HAT (Sigma, MO) and 1% BM-Condimed (Roche). Clone M7 that produces antibodies against the CD169 molecule stably expressed on WR19L cell line (WR-CD169 cells) was selected by flow cytometry. To generate anti-CCL8 antibody, the Wistar rat was immunized subcutaneously in the foot pad with CCL8 peptide (1–13: EKLTGPDKAPVTC) emulsified in adjuvant. Approximately 1,600 hybridomas were established by fusing lymph node cells with myeloma cells as described above. The hybridoma supernatants were tested by ELISA, and hybridomas that specifically detect recombinant CCL8 protein were selected.

### Isolation of LP cells

The digestion of colonic LP was performed based on a method established previously^56^ with slight modification. The entire colon was removed, flushed several times with PBS to remove faeces and opened longitudinally. Two- to three-centimetre pieces of colon were incubated in HBSS supplemented with 2% FBS and 20 mM EDTA, pH 7.2 for 15 min at 37 °C. The tissue was washed in PBS to wash off EDTA, and the residual epithelial layer was gently removed by sliding a spatula. The tissue was minced into 5-mm-long pieces and incubated in RPMI supplemented with 2% FCS, 150 μg ml^−1^ Liberase TL, 500 μg ml^−1^ DNase I (Roche), 1% Dispase (BD Biosciences, CA), 10 mM HEPES (Nacalai, Japan) and 1% penicillin and streptomycin (Invitrogen, NY) for 40 min at 37 °C. The digested pieces were filtered through a 70-μm cell strainer (BD Biosciences) and washed with RPMI/2% FCS/1% penicillin and streptomycin. For the purification of phagocytes, total LP cells were incubated with Fc blocker (BD Biosciences), followed by incubation with CD11c and CD11b microbeads (Miltenyi, Germany). CD11b^+^ and/or CD11c^+^ cells were enriched by magnetic sorting. In some experiments, the cells were further fractionated by a cell sorter (FACS Aria, BD Biosciences or SH800, SONY, Japan). For the purification of mononuclear cells, total LP cells suspended in 1 ml of RPMI/2% FCS/1% penicillin and streptomycin were overlaid on 40 and 80% fractions of Percoll PLUS solution (GE Healthcare, Sweden), and the entire mixture was centrifuged for 25 min at 1,000*g* without brakes. Cells at the interphase were harvested as mononuclear cells.

### Parabiosis surgery

Parabiotic mice were generated using age- and weight-matched female CD169-Cre-YFP (donor) and Rosa26-YFP or CD169-DTR (host) mice based on a previously reported protocol^57^. In brief, a pair of mice was positioned next to each other with their heads pointing in the same direction. Skin incisions that start from the shoulder joint to the ipsilateral hip joint were made. The skin was closed with wound clips. The mice were separated 5 weeks after the surgery. DT was injected into CD169-Cre-YFP-CD169-DTR parabionts 1 week before the separation to deplete CD169^+^ cells.

### DSS-induced colitis

Mice were orally administered with 3.0–3.5% DSS (MW 5,000, Wako, Japan) in drinking water for 7 days, and regular drinking water thereafter. Body weight was monitored daily or every other day for 14 days from the administration of DSS. In some experiments, 25 ng per gram body weight of DT (Sigma) was intraperitoneally injected 1 day before and 3 days after the administration of DSS. For the blocking of CCL8 *in vivo*, 100 μg of anti-CCL8 antibody (clone 17D6, generated in our laboratory, as described previously in the Methods section) or isotype IgG2a (R&D, MN) was intravenously injected on days 3 and 4.

### T-cell-transfer colitis

RAG1 KO mice or RAG1 KO mice crossed with CD169-DTR mice were intravenously injected with 1.6 × 10^5^ CD4^+^, CD45Rb^hi^ naive T cells enriched from spleen and mesenteric LN of WT mice. The mice were injected with DT twice in week 8 to deplete CD169^+^ cells. Body weight change was monitored up to 12 weeks after the adoptive transfer of T cells.

### Histopathology and immunohistochemistry

The distal portion of the colon was fixed in neutralized 10% formalin, embedded in paraffin and stained with haematoxylin and eosin. For the detection of GFP, or TdT-mediated dUTP nick end labelling (TUNEL) staining, tissues were fixed in 4% paraformaldehyde/4% sucrose in 0.1 M phosphate buffer, pH 7.2 for 1 h, sequentially submerged in 10 and 20% sucrose and 50% OCT compound (Sakura Finetek, CA) and snap-frozen in OCT. For the detection of F4/80 or CD169, immunohistochemistry was performed without fixation. Fourteen-μm-thick cryosections were air-dried and rehydrated in PBS. Endogenous peroxidase activity was quenched with 0.8% H_2_O_2_/PBS. Endogenous biotin was blocked with the Biotin Blocking System (Dako, CA) and then with TN blocking buffer (PerkinElmer, MA) or 2% Block Ace (Dainippon, Japan)/1.5% normal goat serum/0.2% Triton X-100/PBS. The sections were stained with biotinylated anti-CD169 (M7), F4/80 (CI:A3-1) or GFP (ab69313) using the TSA Biotin System (Perkin Elmer), and observed under a fluorescent microscope (BZ-8100 or BZ-X700, Keyence, Japan). TUNEL staining was performed according to the manufacturer's protocol using the ApopTag *In Situ* Apoptosis Detection Kit (Millipore, CA). The distance of CX3CR1^+^ or CD169^+^ cells from the epithelial border was quantified by ImageJ software (NIH).

### Flow cytometry

LP cells were incubated with Fc blocker (2.4G2, 2.5 μg ml^−1^) and then stained with a mixture of antibodies, including anti-CD11b (M1/17, 0.5 μg ml^−1^), anti-CD11c (N418, 1.25 μg ml^−1^), anti-CD64 (X54-5/7.1, 2.0 μg ml^−1^), anti-CD103 (M290, 1.0 μg ml^−1^), anti-F4/80 (CI:A3-1, 2.0 μg ml^−1^), biotinylated anti-CD169 (M7, generated in our laboratory, 1.0–4.0 μg ml^−1^), biotinylated anti-CD172a (P84, 1.6 μg ml^−1^), anti-CD206 (C068C2, 2.0 μg ml^−1^), anti-Ly6C (HK1.4, 0.8 μg ml^−1^), anti-Ly6G (1A8, 2.0 μg ml^−1^) and anti-Siglec-F (E50-2440, 2.0 μg ml^−1^). Biotinylated rat IgG2b (RTK4530, 2.0 μg ml^−1^) was used as an isotype control for anti-CD169. Antibodies used for staining LP lymphocytes included anti-CD3ɛ (145-2C11, 2.0 μg ml^−1^), anti-CD4 (L3T4, 0.5 μg ml^−1^), anti-CD45.2 (104, 2.0 μg ml^−1^), anti-B220 (RA3-6B2, 1.0 μg ml^−1^), anti-NKp46 (29A1.4, 4.0 μg ml^−1^) and TCR γδ (GL3, 2.0 μg ml^−1^). 7AAD was added to the cell suspension to exclude dead cells. Cells were analysed by FACS Verse (BD Biosciences).

### Quantitative RT–PCR

Total RNA from colon tissue or LP macrophages was extracted with an RNeasy Mini or Micro kit (Qiagen, the Netherlands) or an RNAspin Mini kit (GE Healthcare) according to the manufacturer's protocol. Complemetary DNAs were synthesized using ReverTra Ace (TOYOBO, Japan). qRT–PCR was performed on complemetary DNA with the THUNDERBIRD SYBR qPCR Mix (TOYOBO). In some experiments, RNA was amplified using WT-Ovation RNA Amplification System (NuGEN, CA). Reactions were run on a real-time PCR system (StepOne Plus, Applied Biosystems, CA). Expression levels were normalized to beta actin or 18 s ribosomal RNA and displayed as fold induction over naive controls, unless otherwise stated. Primer sequences are summarized in Supplementary Table 1.

### Microarray analysis

CD169^+^ and CD169^−^ myeloid cells were purified from the LP of WT naive mice or colitis mice administered with 3.5% DSS for 4 days by a cell sorter (FACS Aria, BD Biosciences). The purities of the fractionated cells were ∼90%. Total RNA extracted from the fractionated myeloid cells was hybridized to Affimetrix Mouse Genome 430 2.0 Array chip. Gene expression heat map was created using Genespring software.

### Establishment of mouse CCL8 ELISA

A 96-well plate (Greiner, Germany) was coated with 10 μg ml^−1^ of anti-CCL8 (MAB790, R&D) in 0.1 M bicarbonate buffer, pH 9.6. After blocking with Assay Diluent (BD Biosciences) for 2 h, macrophage culture medium diluted 2 × with Assay Diluent was applied to each well, and incubation was carried out for 2 h at room temperature. Then, the plate was incubated for 1 h with 1.25 μg ml^−1^ of biotinylated rat IgG anti-CCL8 (clone 12G8, generated in our laboratory) followed by incubation with horseradish peroxidase–streptavidin for 30 min. Finally, substrate solution (TMB Microwell Peroxidase Substrate System, KPL, MD) was added to each well to promote oxidation. After stopping the reaction with 2 M sulfuric acid, optical density at 450 nm was measured with a microplate reader (Bio-Rad, CA).

### Culture of colon explant

Colon from naive or colitis mice was flushed 10 times with PBS to remove faeces. Two 5-mm-long colon tissues were cultured in 250 μl of RPMI/10% FCS/1% penicillin–streptomycin/50 μg ml^−1^ gentamycin (Wako) in a 48-well plate (Corning, NY) at 37 °C for 20 h. CCL8 in the culture supernatant was measured by ELISA.

### *In vitro* macrophage stimulation

BM cells were cultured either in MEMα/10% FCS/10% M-CSF (CMG14-12 culture medium) or in RPMI/10% FCS/10% GM-CSF (MGM-5 culture medium) for 4 days. A total of 1 × 10^5^ cells were seeded in a 96-well plate and stabilized for another 24 h. The cells were stimulated for 24 h either with LPS (*Escherichia coli*, O55:B5, 100 ng ml^−1^, Sigma), ATP (1 mM, Sigma) or HMGB1 (10 μg ml^−1^, Sinotest, Japan). Concentrations of CCL8, IL-6 and TNFα in the culture medium were quantified by ELISA established in our laboratory (CCL8) or an OptEIA ELISA kit (TNFα and IL-6, BD Biosciences).

### Cell migration assay

The chemotactic ability of CCL8 *in vitro* was assessed using a 5-μm-pore transwell system (Corning). WEHI-3 cells (5 × 10^5^) were applied in the upper chamber of the transwell, and 600 μl of serum-free RPMI or serum-free RPMI supplemented with 100 nM of chemokine was applied in the lower chamber to promote migration. After 4 h, the migrated cells in the lower chamber were counted. To assess the chemoattraction of CCL8 *in vivo*, 600 μl of growth factor-reduced Matrigel (BD Biosciences) alone or Matrigel supplemented with 1 μg ml^−1^ of CCL8 was injected subcutaneously into the back of WT mice. Matrigel plugs were resected 24 h after the injection and digested for 90 min with 5 mg ml^−1^ of Collagenase Type IV (Roche). The number of cells retrieved from the Matrigel plugs was quantified by flow cytometry. Nuclear morphology was examined by staining cytospun cells with modified Giemsa (Diff-Quik, Sysmex, Japan).

### Statistical analysis

Data were analysed either by analysis of variance followed by multiple comparison, or by the unpaired *t*-test with Prism (GraphPad Software, CA). Values of *P*<0.05 were considered significant. Least-squares means of the effects of depletion of CD169-positive macrophages or anti-CCL8 administration against body weights were computed by SAS software (SAS Institute, NC).

## Additional information

**Accession codes:** Affymetrix gene expression data were deposited in RefDIC database (http://refdic.rcai.riken.jp) under the following accession numbers: RSM12643, RSM12644, RSM12645, RSM12646, RSM12865, RSM12866, RSM12867 and RSM12868.

**How to cite this article:** Asano, K. *et al.* Intestinal CD169^+^ macrophages initiate mucosal inflammation by secreting CCL8 that recruits inflammatory monocytes. *Nat. Commun.* 6:7802 doi: 10.1038/ncomms8802 (2015).

## Supplementary Material

Supplementary Information Supplementary Figures 1-10 and Supplementary Table 1
