# Supplementary material for: Intestinal CD169+ macrophages initiate mucosal inflammation by secreting CCL8 that recruits inflammatory monocytes
Source: Nat Commun. 2015 Jul 21;6:7802. doi: 10.1038/ncomms8802 (PMC4518321; doi:10.1038/ncomms8802)
Supplement: Supplementary Information — Supplementary Figures 1-10 and Supplementary Table 1 [file ncomms8802-s1.pdf]

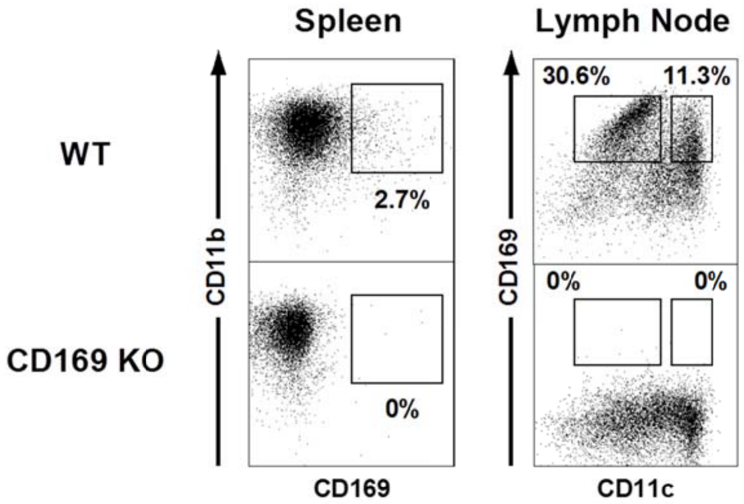

Supplementary Figure 1. Specificity of anti-CD169 antibody, clone M7.
CD11b$^+$ cells from spleen (left) or lymph node (right) of WT (top) or CD169 KO (bottom) mice were enriched by magnetic sorting. The cell suspension was stained with anti-CD169 and anti-CD11b (spleen) or anti-CD11c (lymph node) antibodies. Numbers indicate the frequency of CD169$^+$ cells among 7AAD$^-$ fraction. Representative of 2 independent experiments.

Supplementary Figure 1

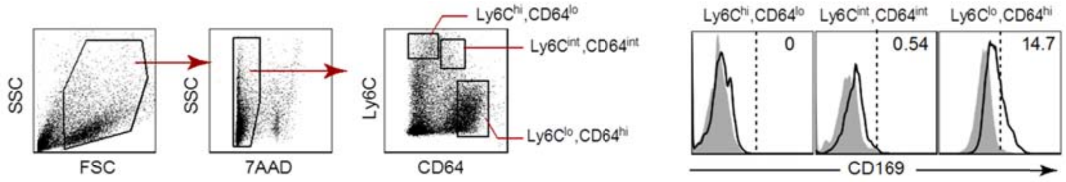

Supplementary Figure 2. Expression of CD169 on the LP cell of WT small intestine.
LP myeloid cells were enriched from the small intestine of WT mice and stained for Ly6C and CD169. Shadow represents isotype control. Represenative data of 3 independent experiments.

Supplementary Figure 2

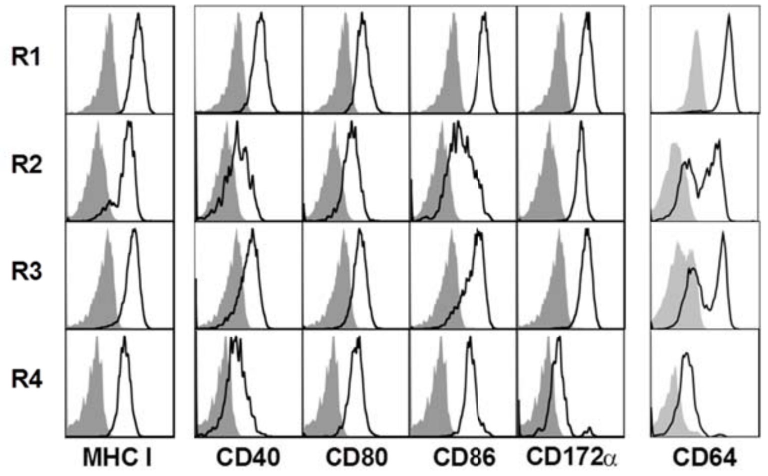

Supplementary Figure 3. Phenotypic characterization of LP CD169$^+$ macrophages of the colon. Flowcytometry of LP CD169$^+$ and CD169$^-$ cells. LP myeloid cells were enriched from WT mice by magnetic sorting and gated according to the expression of CD169, CD11b and CD11c (R1-R4, as described in **Fig. 1C**) . Dead cells were excluded by 7AAD. Shadow represents unstained control. Representative of 2 independent experiments.

Supplementary Figure 3

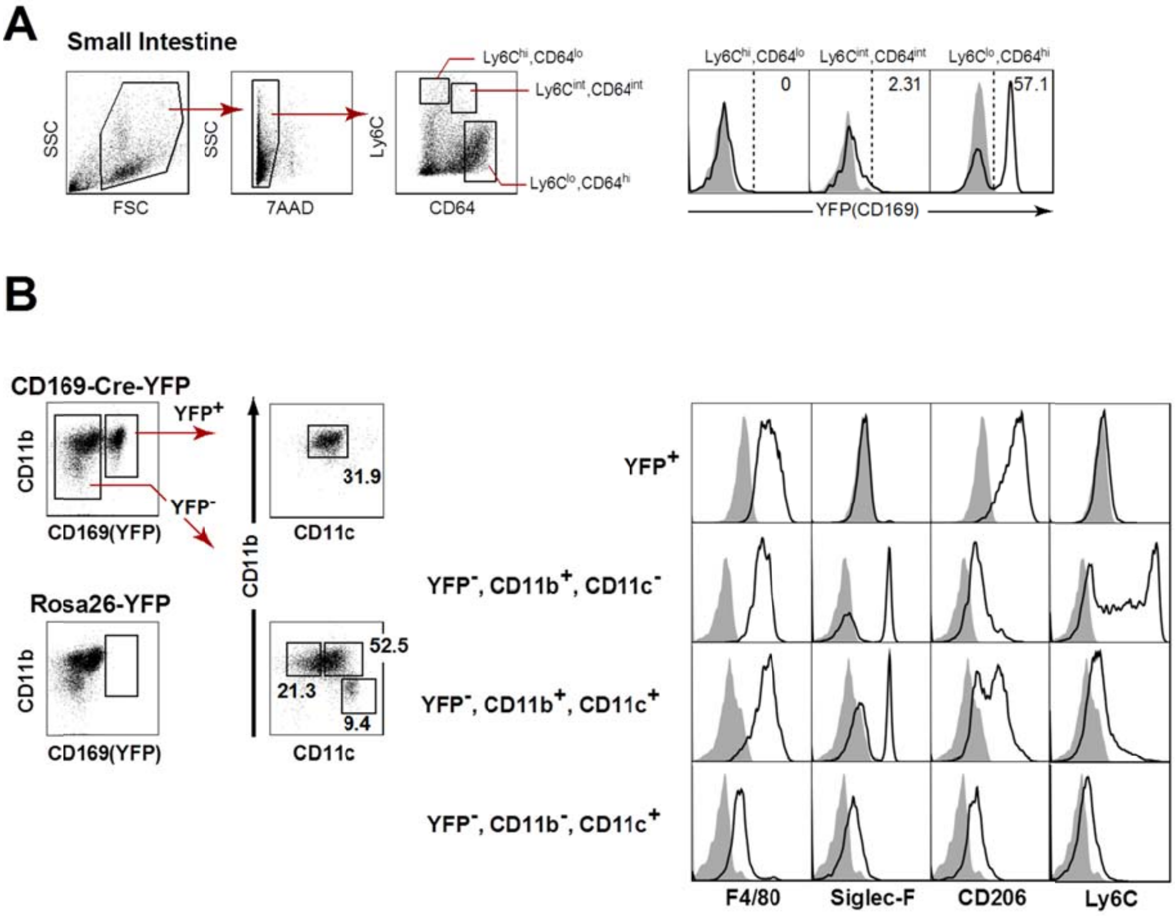

Supplementary Figure 4. Phenotypic characterization of CD169 (YFP)-positive and -negative cells in the LP of CD169-Cre-YFP mice.

(A) Flowcytometric analysis of LP myeloid cells of the small intestine of CD169-Cre-YFP mice. CD11b^{+} and/or CD11c^{+} cells were enriched by magnetic sorting. Those cells were stained for Ly6C and CD64, and analyzed by a flowcytometer. Dead cells were excluded by 7AAD. Numbers indicate the frequencies of CD169^{+} cells among each fraction. Shadows indicate WT controls.

(B) Flowcytometric analysis of LP myeloid cells of the colon of CD169-Cre-YFP mice. Rosa26-YFP mice do not express YFP (bottom left). CD11b^{+} and/or CD11c^{+} cells enriched by magnetic sorting were stained for CD11b, CD11c, CD169 and indicated surface molecules. Dead cells were excluded by 7AAD. Shadows indicate unstained controls. Representative data of 3 independent experiments.

Supplementary Figure 4

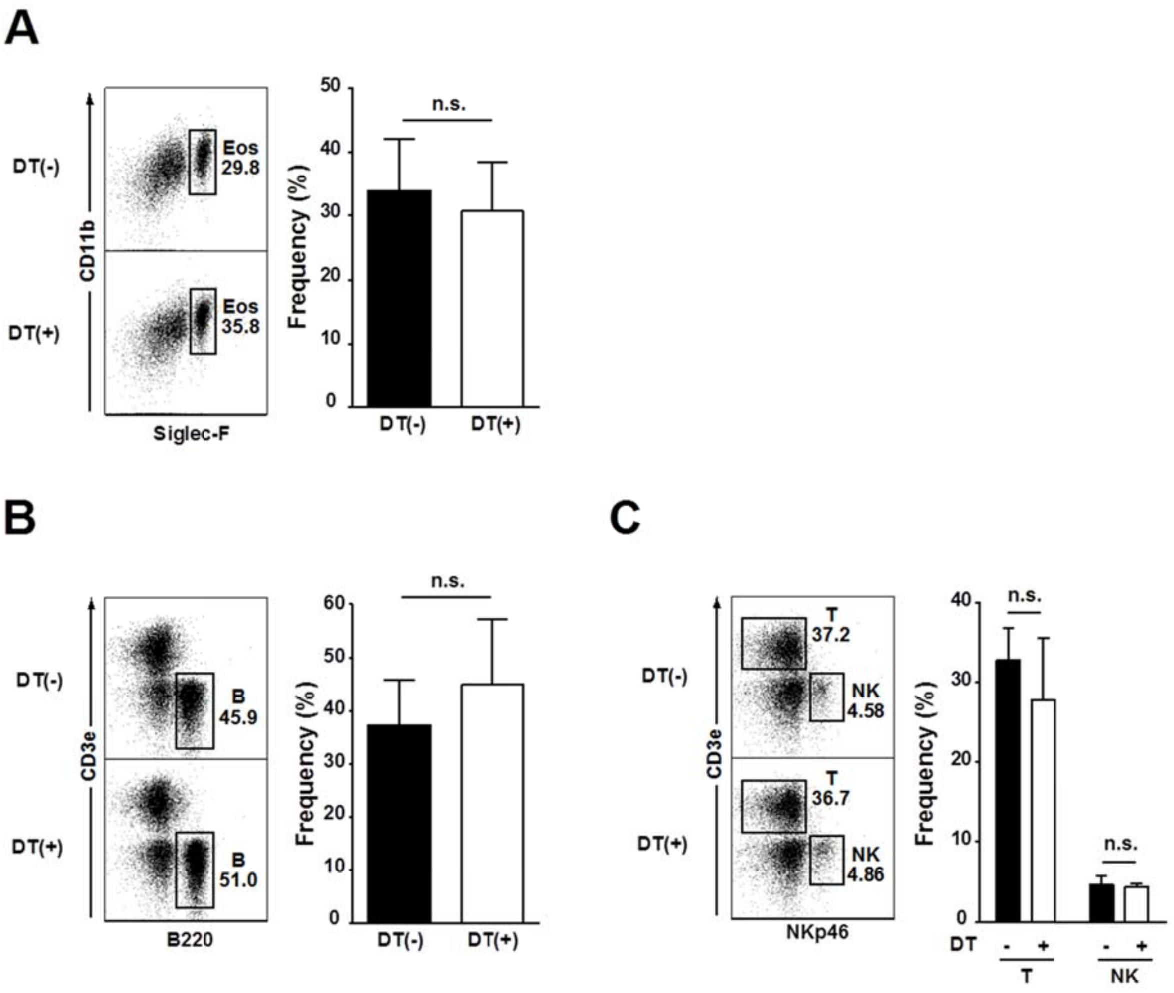

Supplementary Figure 5. Selective depletion of CD169^{+} cells in CD169-DTR mice.

(A) DT injection into CD169-DTR mice does not deplete eosinophils.

Flowcytometric analysis of LP myeloid of CD169-DTR mice. LP CD11b^{+} and/or CD11c^{+} cells were enriched by magnetic sorting from CD169-DTR mice 1 day after injection of PBS or DT and stained for CD11b and Siglec-F. Average values and s.d. of 3 mice are shown.

(B) and (C) DT injection into CD169-DTR mice does not deplete lymphocytes in the LP. Flowcytometric analysis of LP lymphocytes of CD169-DTR mice. LP cells from CD169-DTR mice 1 day after DT injection were purified by Percoll density gradient and stained for CD3e, CD45.2, B220, and NKp46. Frequencies of B cells (B), T and NK cells (C) did not change by DT injection. Average values and s.d. of 3 mice are shown. Numbers indicate percentage among CD45^{+}, 7AAD^{-} cells. n.s., not significant.

Supplementary Figure 5

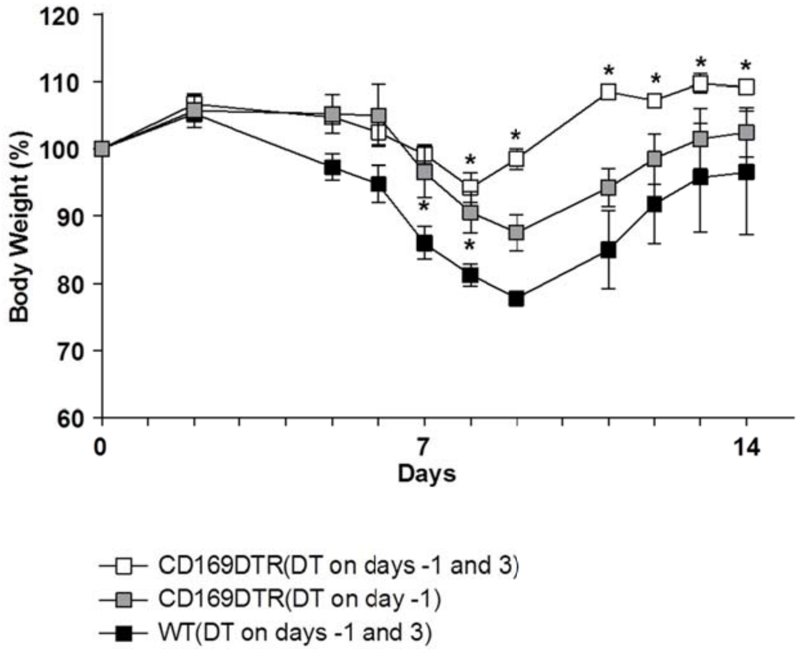

Supplementary Figure 6. Single injection of DT was sufficient to protect CD169-DTR mice from DSS-induced colitis.

WT mice were administered with DT on days -1 and 3 (black square). CD169-DTR mice were administered with DT either on day -1 alone (gray square) or on days -1 and 3 (white square). Those mice were administered orally with 3.5 % DSS for 7 days. *, $p<0.05$, two-way ANOVA with multiple comparison. n=4 mice per group. Representative data of 2 independent experiments.

Supplementary Figure 6

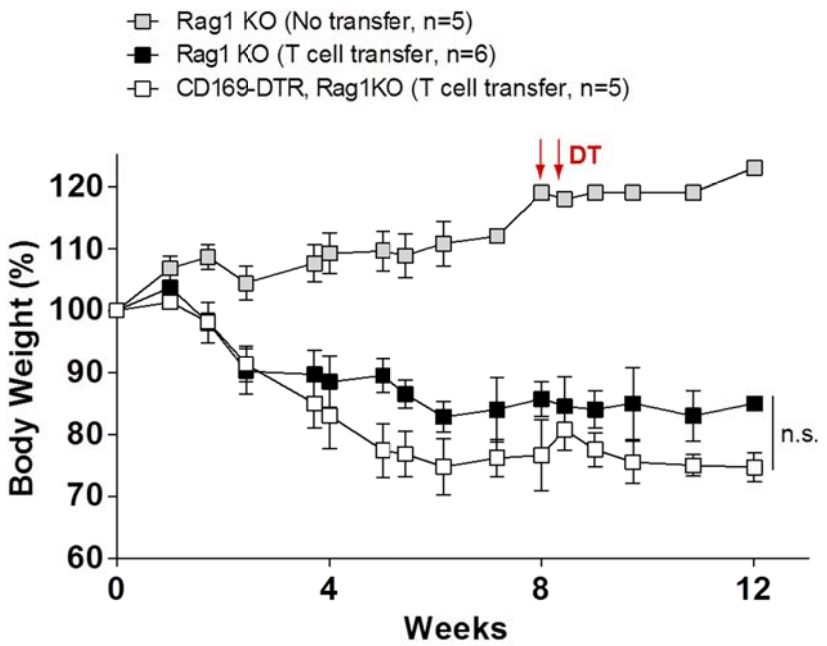

Supplementary Figure 7. CD169⁺ macrophageds are dispensable for the development of T cell-transfer colitis.

Rag1 KO mice or RAG1 KO mice that were crossed with CD169-DTR mice were adoptively transferred with 1.6 x 10^5 WT naive CD4 T cells. Those mice were administered with DT twice in week 8 (red arrows) to deplete $CD169^+$ cells. n.s., not significant.

Supplementary Figure 7

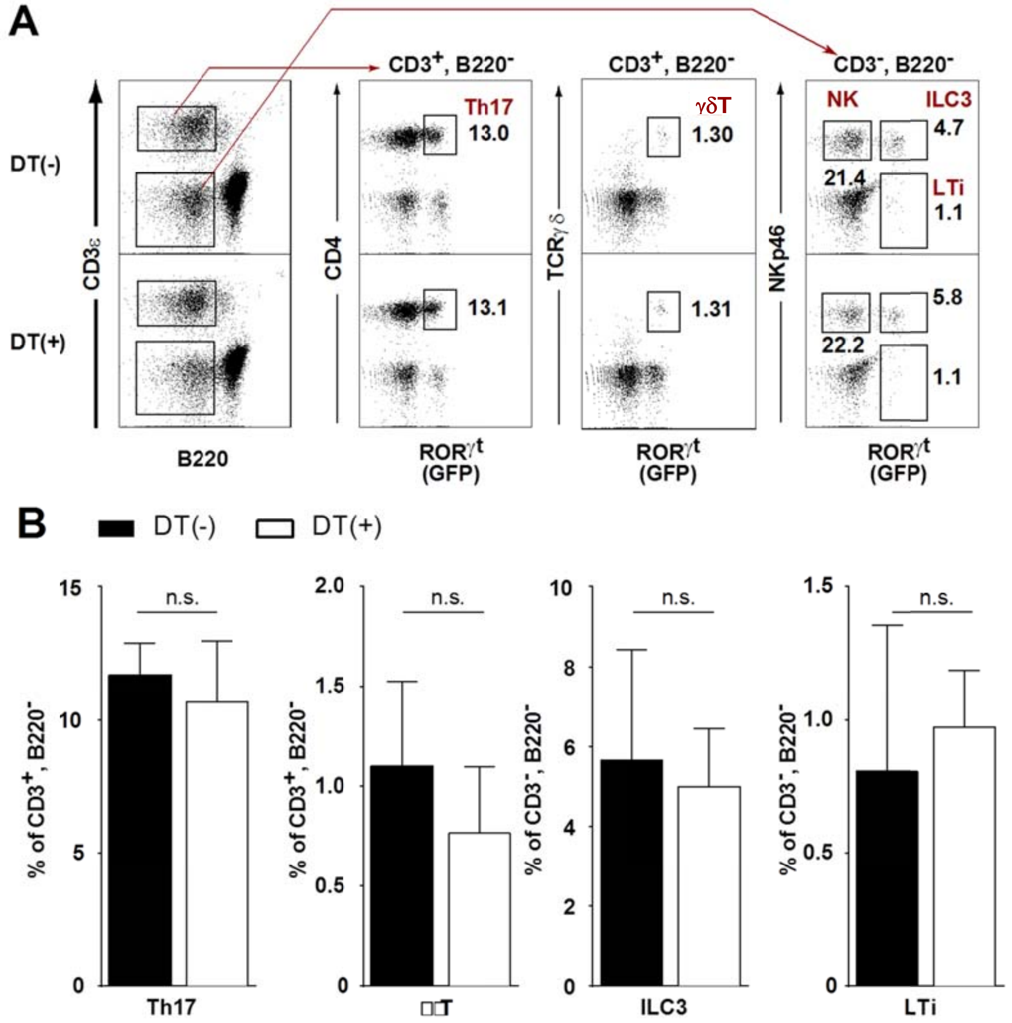

Supplementary Figure 8. DT injection does not deplete innate lymphoid cells in the LP of the colon of RORγt^{gfp}, CD169-DTR mice.

RORγt^{gfp}, CD169-DTR mice were administered orally with 3.5 % DSS for 7 days. Those mice were injected with PBS or DT on days -1 and 3. (A) Frequencies of producers of Th17 cytokine family were analyzed by flowcytometry on day 7. (B) Average values and s.d. of 3 mice per group are shown with s.d.. n.s., not significant, Student' s t-test.

Supplementary Figure 8

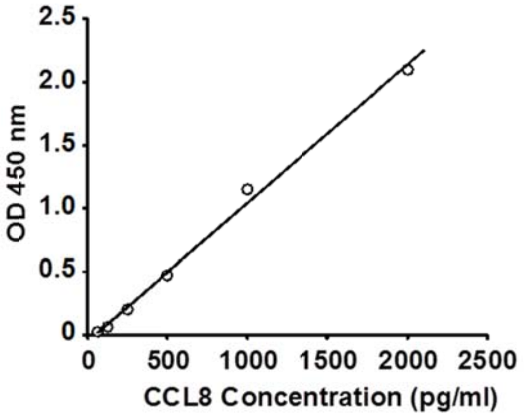

Supplementary Figure 9. Establishment of mouse CCL8 ELISA.

The standard dose-response curve exhibited a linear shape in the concentration range of 50 pg/ml to 2 ng/ml.

Supplementary Figure 9

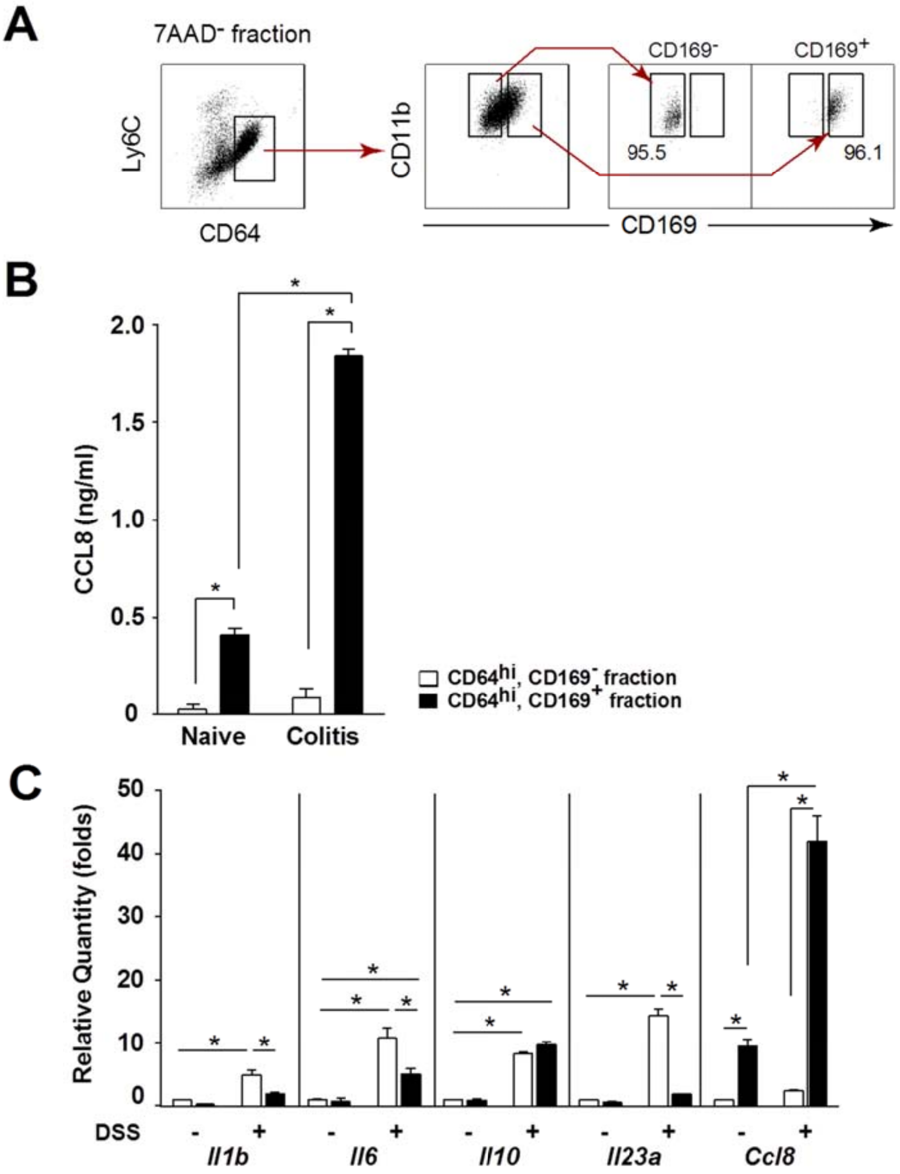

Supplementary Figure 10. Production of CCL8 and other pro- and anti-inflammatory cytokines by LP CD64^{hi} macrophages in the colon.

LP CD11b^{+} cells from WT naive or colitis mice were enriched by magnetic sorting. CD64^{hi}, CD169^{+} and CD64^{hi}, CD169^{-} cells were further fractionated by a cell sorter. The purity of fractionated cells was above 95 % (A). Those cells were seeded on a 96-w flat bottom plate at a concentration of 1 x 10^{5} cells/w and culturefd for 20 h at 37 °C. Concentrations of CCL8 were quantitated by ELISA (B). Cytokine mRNA expression levels in fractionated cells were quantitated by qRT-PCR (C). $p<0.05$, two-way ANOVA. Average values and s.d. of triplicate experiment.

Supplementary Figure 10

Supplementary Table 1 Sequences of quantitative RT-PCR primers

Genes		Sequence(5'→3')
Rn18s	Fwd	CGGACAGGATTGACAGATTG
	Rev	CAAATCGCTCCACCAACTAA
Il1b	Fwd	GGATGAGGACATGAGCACCT
	Rev	AGCTCATATGGGTCCGACAG
Il6	Fwd	CTGGAGTACCATAGCTACC
	Rev	CTGTTAGGAGAGCATTGGA
Il23a	Fwd	CAGGGAACAAGATGCTGGAT
	Rev	GGCTAGCATGCAGAGATTCC
Tnf	Fwd	ACCCTCACACTCAGATCATC
	Rev	GAGTAGACAAGGTACAACCC
Ccl8	Fwd	GCTGTGGTTTTCCAGACCAA
	Rev	GAAGGTTCAAGGCTGCAGAA
Il10	Fwd	CCAAGCCTTATCGGAAATGA
	Rev	TTTTCACAGGGGAGAAATCG
Il17	Fwd	CTCCAGAAGGCCCTCAGACTAC
	Rev	AGCTTTCCCTCCGCATTGACACAG
Il22	Fwd	GCTCAGCTCCTGTCACATCA
	Rev	CAGACGCAAGCATTTCTCAG
